# Depolymerization as a Design Strategy: Depolymerization Etching of Polymerization-Induced Microphase Separations

**DOI:** 10.1021/acscentsci.5c01313

**Published:** 2025-10-29

**Authors:** Kaden C. Stevens, Megan E. Lott, Kiana A. Treaster, Robert M. O’Dea, Adarsh Suresh, Cabell B. Eades, Victoria L. Thompson, Jared I. Bowman, James B. Young, Austin M. Evans, Stuart J. Rowan, Thomas H. Epps, Brent S. Sumerlin

**Affiliations:** † George and Josephine Butler Polymer Research Laboratory, Center for Macromolecular Science & Engineering, Department of Chemistry, 3463 University of Florida, Gainesville, Florida 32611, United States; ‡ Department of Chemical and Biomolecular Engineering, 5972 University of Delaware, Newark, Delaware 19716, United States; § Center for Plastics Innovation (CPI), 5972 University of Delaware, Newark, Delaware 19716, United States; ∥ Center for Research in Soft matter and Polymers (CRiSP), 5972 University of Delaware, Newark, Delaware 19716, United States; ⊥ Prtizker School of Molecular Engineering, 2462 The University of Chicago, Chicago, Illinois 60637, United States

## Abstract

Thermally triggered depolymerization has traditionally been viewed through the lens of sustainability and recycling, not as a constructive tool for materials design. Herein, we show that selective, thermally triggered depolymerization to gaseous monomer serves as a solvent-free strategy for generating porosity in nanostructured polymer materials, offering a means to bypass the mass transport limitations inherent in conventional solution-based etching. As a demonstration platform, we employed polymerization-induced microphase separation (PIMS) to generate disordered bicontinuous block copolymer structures with embedded depolymerizable domains. By incorporating a methacrylate block susceptible to thermal depolymerization within a cross-linked, depolymerization-resistant styrenic matrix, we developed a process we term depolymerization etching of polymerization-induced microphase separations (DEPIMS). This approach enables highly selective and efficient domain removal via reversion to monomer to produce mesoporous materials with high surface areas (>200 m^2^/g). Subsequent surface functionalization yielded mesoporous adsorbents with tunable uptake kinetics and among the highest dye adsorption capacities reported for PIMS-derived materials, demonstrating the adaptability of the DEPIMS platform for chemical separations. DEPIMS can also be extended to a gram-scale, one-pot approach to yield mesoporous materials with recoverable monomer in under 12 h. These findings reposition thermal depolymerization from a sustainability tool to a broadly enabling strategy for scalable, on-demand fabrication of functional nanostructured materials.

## Graphical abstract

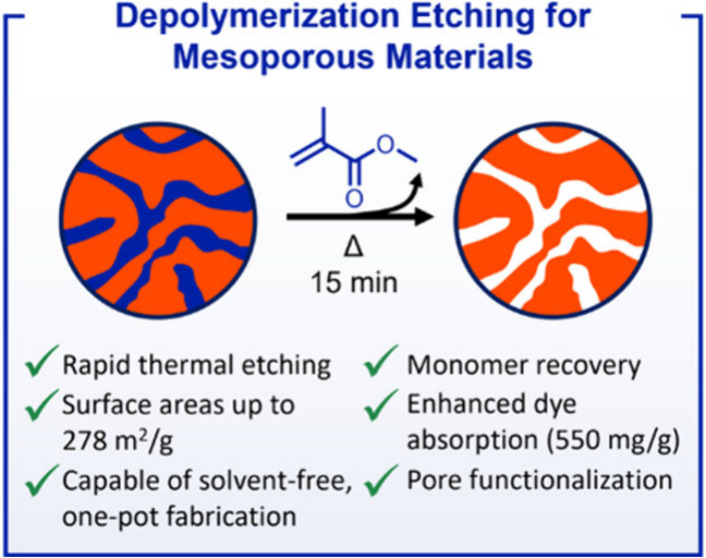

## Introduction

Depolymerization has traditionally been viewed as a strategy for polymer degradation or chemical recycling.^1–10^ However, recent developments indicate that depolymerization efficiency is highly sensitive to backbone chemistry,^11–14^ which suggests opportunities for this process to be used as a means to constructively remove targeted domains within multicomponent materials. We envisioned that selective, thermally triggered depolymerization from bulk polymer to gaseous monomer could serve as a solvent-free approach to generate nanostructured polymer systems. In particular, we reasoned that recent advances in vinyl depolymerization^11,15,16^ could be leveraged to generate polymers capable of on-demand deconstruction under relatively mild conditions.

We have developed an efficient approach toward bulk depolymerization whereby a small quantity of comonomer with thermolytically labile pendent groups is embedded within a vinyl copolymer.^11,16–18^ At moderate temperatures, these pendent groups are liberated from the backbone to reveal tertiary radicals that lead to polymer backbone fragmentation and result in radicals capable of depropagation. Crucially, pendent-group initiated depolymerization processes require significantly lower temperatures (250–300 °C) than traditional pyrolytic depolymerization approaches (400–600 °C), which reduces undesirable side-reactions, leading to higher monomer purity and recovery.^19^ However, depolymerization is not equally effective for all vinyl polymers. For example, embedding 1 mol% of the thermolytically labile trigger *N*-(methacryloxy)­phthalimide methacrylate (PhthMA) as a comonomer within a poly­(methyl methacrylate) (PMMA) copolymer resulted in >90% reversion to monomer at 290 °C over 15 min, whereas the same 1 mol % PhthMA incorporation within a polystyrene (PSty) copolymer resulted in <10% reversion to monomer when subjected to 290 °C for 2 h.^11,16^

While the different depolymerization efficiencies of styrenic and methacrylic backbones suggest promising selectivity for etching applications, the high temperatures required for bulk thermal depolymerization present opportunities and challenges. One distinct advantage is that these elevated temperatures often exceed the boiling point of the liberated monomer, which enables facile removal of depolymerization products as gaseous species.^16^ The release of rapidly diffusing gas upon depolymerization presents an opportunity to dramatically reduce the etching time for challenging substrates like large-scale nanostructured monoliths, as standard, solution-based approaches are inherently limited by slow inward diffusion of the etchant and outward diffusion of degradation products.^20,21^ However, depolymerization temperatures for bulk materials are also substantially higher than the glass transition temperature (*T*_g_) of most polymers, which could compromise the structural and morphological stability of the nanostructured materials generated upon etching.^22^ This challenge inspired our design of cross-linked monoliths that would retain their nanostructure following domain-specific removal.

We selected polymerization-induced microphase separation (PIMS)^23,24^ as a versatile and scalable platform through which the advantages of this depolymerization-driven etching process could be realized. PIMS is a straightforward, one-step fabrication process capable of producing high quantities of nanostructured block copolymer materials in bulk, making PIMS an attractive alternative to the multistep synthesis, purification, and processing approaches typically used to generate self-assembled block copolymer structures.^20,23,24^ The PIMS process relies on chain extension of a macroinitiator in the presence of mono- and multifunctional monomers, which results in simultaneous chain extension, microphase separation, and cross-linking that freezes the growing block copolymers into a disordered bicontinuous state. The structural features of the cocontinuous domains within PIMS materials can be controlled via copolymer design to generate well-defined pore sizes and domain volumes.^25–27^ Additionally, domain-selective etching of PIMS materials can reveal mesoporous structures distributed isotropically, with highly connected pores possessing high surface areas, making them attractive materials for catalysis, battery, and purification applications (Figure 1).^28–32^ Despite their promise, porous PIMS materials remain underutilized, as most etching approaches suffer from some combination of stringent macroinitiator synthesis, challenging end-group modification prior to chain extension, and slow solution-based etching techniques.^32–34^ Ideally, rapid, scalable methods for selectively removing one phase without damaging the overall structure could be harnessed via a robust synthetic approach to generate porous PIMS materials.

**Figure 1:**
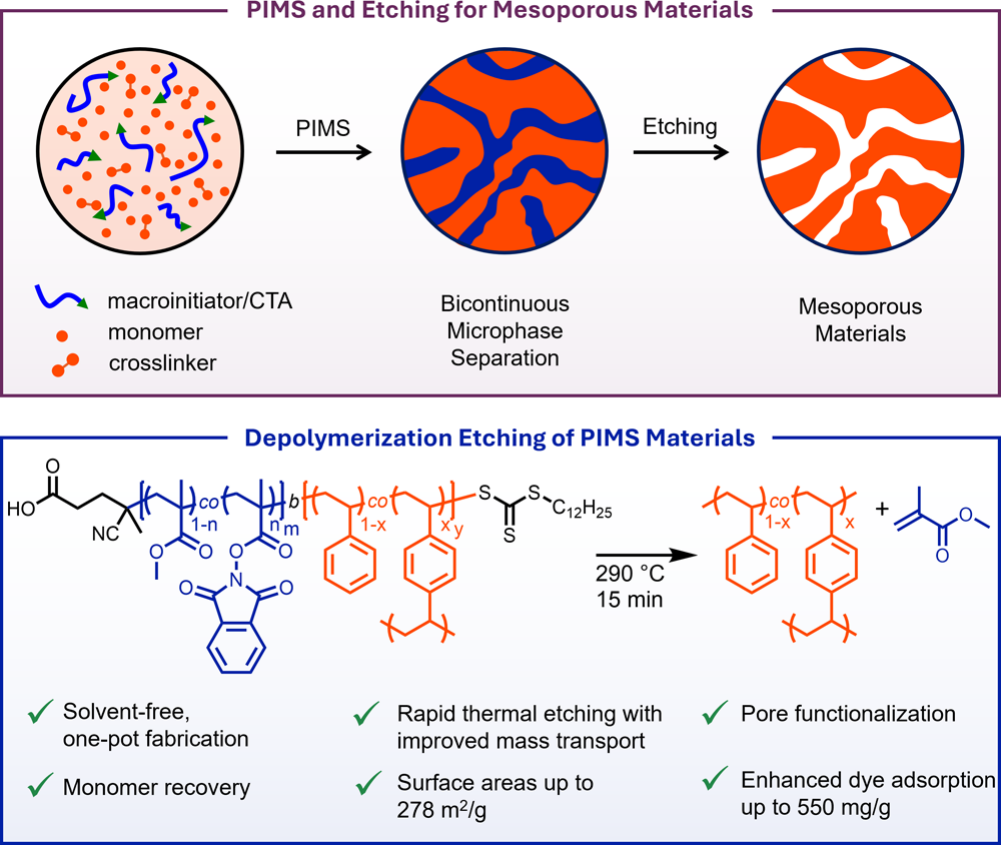
Representative structures, processing conditions, and attributes of materials obtained via (top) hydrolytic etching of polymerization-induced microphase separation (PIMS) materials and (bottom) depolymerization etching of polymerization-induced microphase separations (DEPIMS).

We hypothesized that differential depolymerization tendencies of methacrylic and styrenic backbones would yield a selectively etchable matrix that would ultimately generate mesoporous constructs in a new process we refer to as depolymerization etching of polymerization-induced microphase separations (DEPIMS, Figure 1). We reasoned that poly­(MMA-*co*-PhthMA) (P­(MMA-*co*-PhthMA)) could serve as a readily depolymerizable macro-chain transfer agent (macroCTA) while a cross-linked matrix composed of Sty and divinylbenzene (DVB) could resist depolymerization and maintain structural integrity at elevated temperatures. Importantly, this approach would eliminate the need for demanding polymerization conditions and intermediate chain-end functionalization while also leveraging rapid diffusion of the liberated gaseous monomer to overcome the slow diffusion of etching solutions and degradation products currently required to generate mesoporous PIMS materials.

## Results and Discussion

To establish a depolymerizable domain, we synthesized a copolymer of PhthMA and MMA via reversible addition–fragmentation chain-transfer (RAFT) polymerization. The resulting copolymer macroCTA contained 8 mol % PhthMA, as determined by ^1^H NMR spectroscopy (Figure S1). Size-exclusion chromatography coupled with multiangle light scattering (SEC-MALS) revealed a unimodal peak with a number-average molecular weight (*M*_n, SEC_) of 40.5 kg/mol and a dispersity (*Đ*) of 1.03 (Figure S2). To create materials via PIMS, P­(MMA-*co*-PhthMA) was dissolved at 30 wt % in mixtures of Sty and DVB, with DVB comprising 30, 50, 70, or 100 mol % of the styrenic mixture (Figure 2A). We chose to incorporate P­(MMA-*co*-PhthMA) at 30 wt % into the PIMS materials to generate high surface areas while ensuring the styrenic network remained resilient at the high temperatures required for thermolytic PhthMA-initiated depolymerization (>250 °C). The dissolved mixture was sparged with argon for 5 min and heated to 120 °C for 5 h to cure the mixture via PIMS during the chain extension of P­(MMA-*co*-PhthMA) with Sty and DVB. For brevity, materials generated via the PIMS process will be referred to as PIMS_*x*_, and the fabricated nanoporous materials etched via DEPIMS will be labeled as DEPIMS_*x*_, wherein x denotes the mol % of DVB present in the styrenic portion of the material.

**Figure 2:**
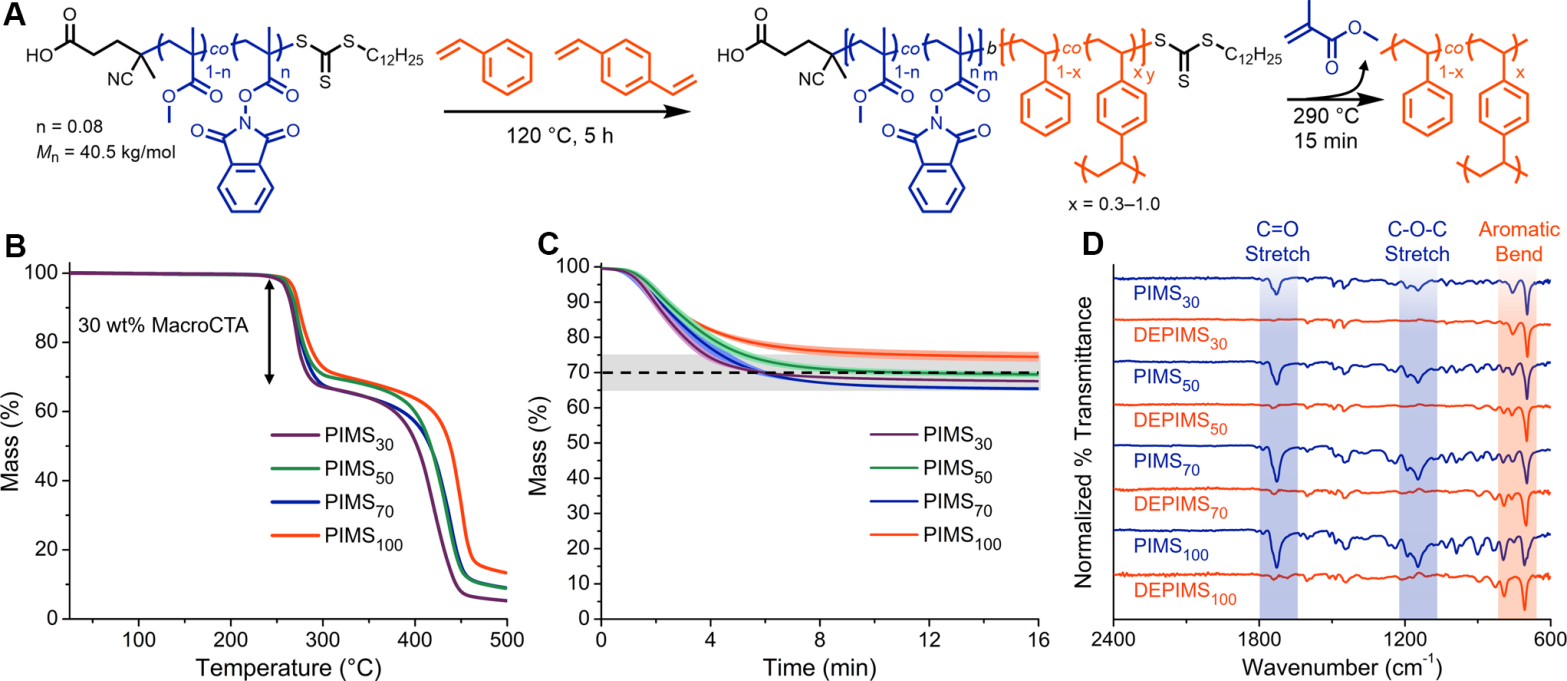
(A) Reaction scheme for PIMS and DEPIMS processes. (B) Thermogravimetric analysis (TGA) temperature sweeps of PIMS materials with varying cross-link densities in the styrenic regions. (C) TGA isothermal holds at 290 °C for 15 min. The dashed line indicates the 30% mass attributable to depolymerization of the P­(MMA-*co*-PhthMA) within the PIMS materials, and the shaded region denotes a ±5% variability around the expected 30% mass loss value. (D) FTIR spectra for PIMS and DEPIMS demonstrating the removal of P­(MMA-*co*-PhthMA) upon depolymerization. Regions shaded in blue are associated with the methacrylic portions of the material, and regions shaded in orange are associated with the styrenic regions.

We then set out to assess the thermal deconstruction behavior of the PIMS materials. Thermogravimetric analysis (TGA) of PIMS materials revealed two prominent mass loss events (Figure 2B). The PIMS materials lost approximately 30% of their mass at 250–300 °C during a heating ramp at 5 °C/min, which corresponded to the mass incorporation of the sacrificial macroCTA within each sample. After a mass loss plateau, another mass loss event occurred at 400 °C, corresponding to degradation of the styrenic portions of the microphase-separated materials. The PIMS_50_ materials were additionally subjected to isothermal holds at a range of temperatures from 260 to 300 °C for 15 min (Figure S3) to determine the optimal temperature for depolymerization. We chose 290 °C as the DEPIMS processing temperature for this formulation as it balanced speed and selectivity of the depolymerization etching process. For all PIMS materials, mass losses during isothermal holds at 290 °C for 15 min were consistent (standard deviation <1.03 wt %) and resulted in mass losses corresponding to the weight of macroCTA incorporation in the PIMS to within 5 wt % (Figure 2C). Notably, the mass loss of the PIMS materials held at 290 °C plateaued after approximately 8 min, suggesting the removal of gaseous monomer from the nanoporous channels was extremely rapid. FTIR spectroscopy of the selectively depolymerized materials revealed efficient removal of the polymethacrylate blocks, as evidenced by the attenuation of signals at 1720 and 1150 cm^–1^, corresponding to the methacrylic C=O stretch and C–O–C stretch, respectively, relative to the aromatic C–H out-of-plane bend from the polystyrenic block at 700 cm^–1^ (Figure 2D). Importantly, PIMS materials generated with a PMMA homopolymer as the macroCTA showed very little mass loss until 400 °C (Figures S4 and S5).

TGA tandem mass spectrometry (TGA-MS) revealed a high selectivity for MMA removal relative to the polystyrenic block during depolymerization. We examined the off-gassed products via TGA-MS at 290 and 450 °C to determine what products were formed during the two mass loss events shown in Figure 2C. TGA-MS confirmed selective depolymerization of the polymethacrylate domain at 290 °C, with minimal Sty evolution and no detectable signals from DVB, offering evidence of the chemical orthogonality central to the DEPIMS strategy. In contrast, MS analysis at 450 °C revealed very little mass loss corresponding to MMA but large mass loss signals corresponding to Sty, DVB and its impurities, and fragmentation byproducts such as 1-methyl-4-vinylbenzene and 1-ethyl-4-vinylbenzene (Figure S6). With confirmation that MMA, Sty, and DVB were the major deconstruction products, we chose to focus on these three molecules under various thermolytic conditions (Figure 3).

**Figure 3:**
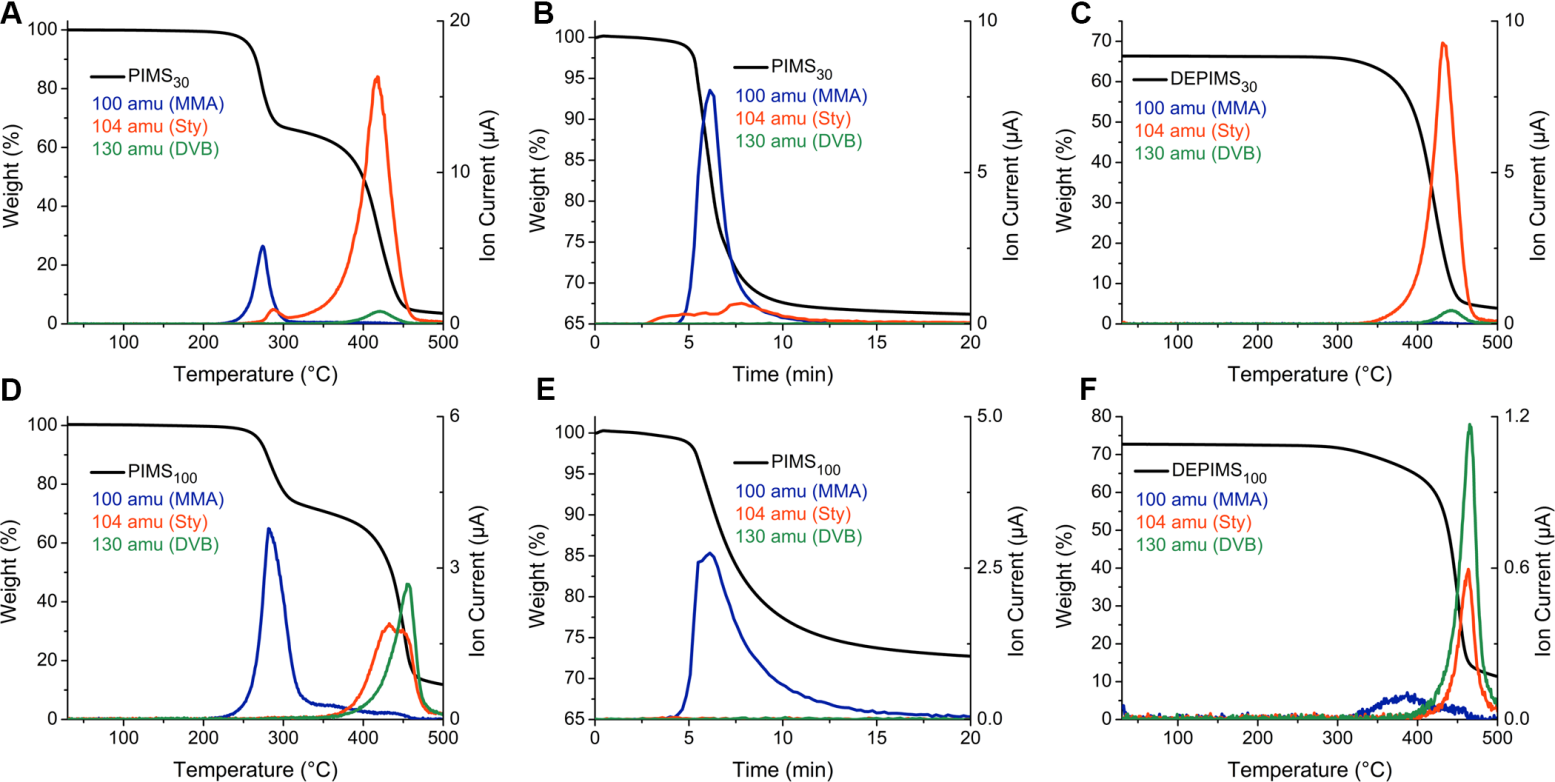
TGA tandem mass spectrometry (TGA-MS) thermograms of PIMS (A and D) taken from room temperature to 500 °C at 5 °C/min, (B and E) held at 290 °C for 15 min, and (C and F) taken from room temperature to 500 °C at 5 °C/min. Black traces are mass loss. Ion current from MS of PIMS is indicated by blue traces for MMA, orange traces for Sty, and green traces for DVB.

TGA-MS ramps to 500 °C at 5 °C/min of PIMS_30_ revealed that mass loss at 250–300 °C was primarily a result of MMA liberation, with a small fraction of Sty and no detectable DVB (Figure 3A,D and Figure S7). Beyond 300 °C, Sty became the primary off-gas of DEPIMS_30_ materials, with no detectable MMA evolved. This selectivity of depolymerization exhibited an interesting trend as cross-linking density increased, where depolymerization in the 250–300 °C range became progressively more selective toward MMA relative to the styrenics. Depolymerization efficiency decreased as DVB content increased, as revealed by more MMA being detected later in the temperature ramp in the 300–450 °C range. The increase in depolymerization selectivity likely emerged from the challenges associated with evolving DVB relative to Sty, since DVB release requires two successful depropagation events to be released. We hypothesize the decreased efficiency of MMA removal could arise from a diffuse interface between the polymethacrylic and polystyrenic domains generated by rapid cross-linking at high DVB content. This blurred phase boundary could generate kinetic barriers or promote side reactions that inhibited the complete depolymerization of P­(MMA-*co*-PhthMA).

The selectivity and effectiveness of the DEPIMS process were further investigated by subjecting the PIMS materials to isothermal holds at 290 °C for 15 min, followed by a ramp to 500 °C at 5 °C/min (Figure 3C–F and Figures S8 and S9). As cross-linking density increased, selectivity during the isothermal hold increased, with noticeably less Sty being evolved from DEPIMS_100_ compared to DEPIMS_30_. However, higher cross-link density led to more residual MMA being evolved from DEPIMS_100_ compared to DEPIMS_30_ during the ramp to 500 °C, suggesting that 15 min at 290 °C resulted in a small amount of residual polymethacrylate within DEPIMS_100_ materials but no detectable polymethacrylate within DEPIMS_30_.

Confident that we had identified DEPIMS fabrication conditions that resulted in selective and rapid depolymerization of the polymethacrylate block, we then characterized the structure of the resulting porous DEPIMS materials via scanning electron microscopy (SEM) (Figure 4 and Figures S10–S13). SEM revealed mesoporous monoliths with morphologies dependent on cross-link density (Figure 4A). At low cross-link density (DEPIMS_30_), insufficient mechanical reinforcement led to pore collapse during thermal processing, highlighting the importance of cross-link architecture in structural retention. In contrast, the DEPIMS materials with higher cross-link densities exhibited clear mesoporous voids consistent with selective etching of one domain within a cocontinuous microstructure. At moderately high DVB content, DEPIMS_50_ and DEPIMS_70_ materials exhibited a skeletal frame of interlaced globules. By contrast, DEPIMS_100_ materials displayed a compact array of spheroidal nodules with less distinct voids, likely due to extensive cross-linking arresting phase separation at low monomer conversion in the materials with polystyrenic blocks comprised of 100% DVB. This morphological evolution as a function of cross-linker content is consistent with previous reports of PIMS being employed to prepare mesoporous materials.^25^ Interestingly, at higher DVB incorporations, the DEPIMS surfaces become noticeably rougher, likely due to high cross-link density at early reaction times inhibiting the formation of sharp interfaces.

**Figure 4:**
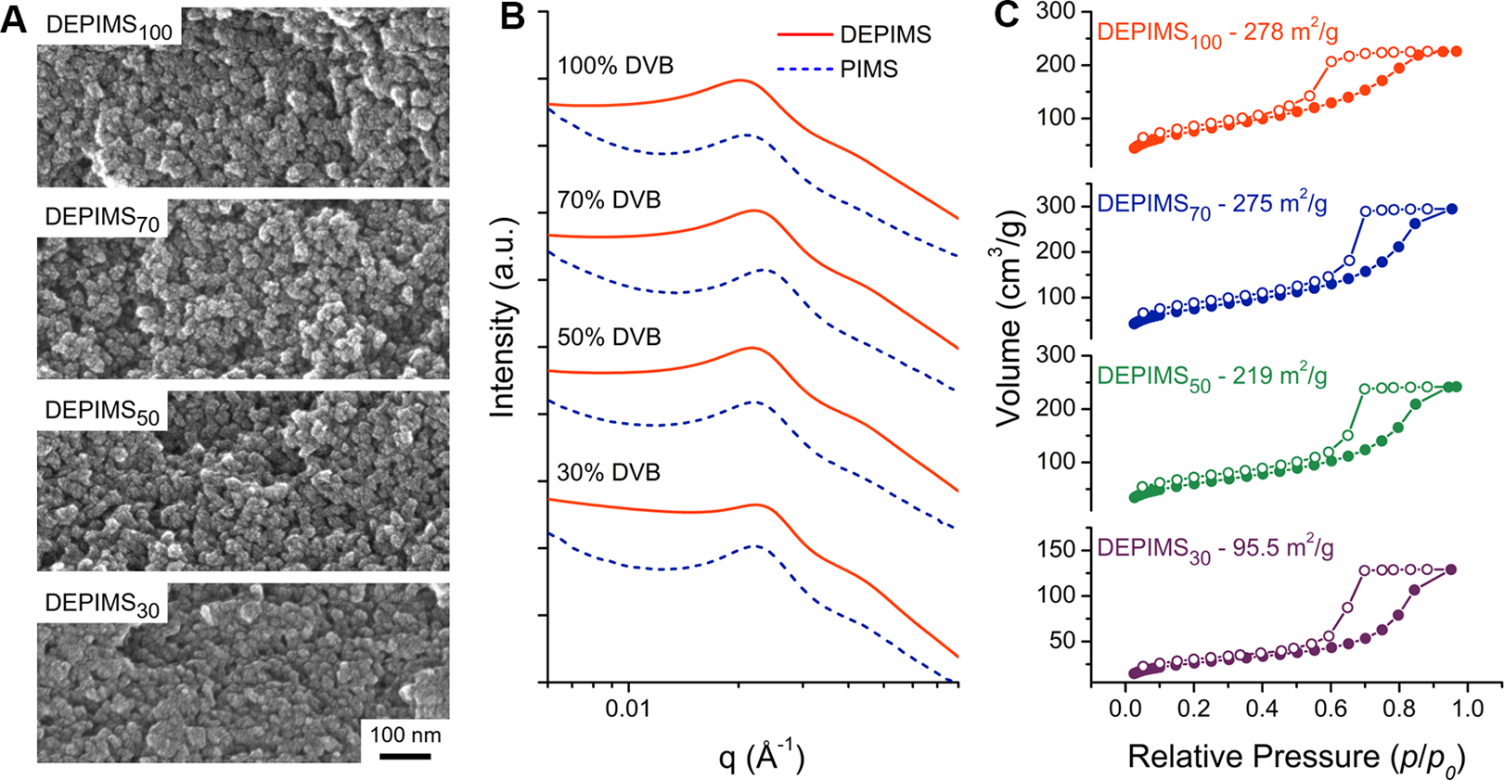
Structural characterization of DEPIMS materials. (A) Scanning electron micrographs of DEPIMS demonstrating the loss of porosity at low cross-linking densities (DEPIMS_30_) and pore retention at higher DVB content. (B) Small-angle X-ray scattering (SAXS) patterns from PIMS and DEPIMS at each DVB content that indicate structure is lost at low cross-linking densities (DEPIMS_30_). SAXS patterns are vertically shifted for clarity. (C) Nitrogen sorption isotherms with Brunauer–Emmett–Teller (BET) surface areas suggesting surface area is lost because of low cross-linking density for DEPIMS_30_. Details of BET surface area fitting can be found in Table S1. Adsorption and desorption isotherms are indicated by filled and unfilled circles, respectively.

Small-angle X-ray scattering (SAXS) analysis of the PIMS materials indicated the characteristic structure factor peaks q* and 2q* expected for the disordered bicontinuous morphology characteristic of most materials prepared by PIMS (Figure 4B). The position of the q* peak at approximately 0.022 Å^–1^ indicated an average domain spacing (*d*) of 28.5 nm via the relation *d* = 2π/*q*, which is reasonable for a macroCTA of 40.5 kDa when compared to previous reports.^20^ After etching, the DEPIMS monoliths retained the same domain spacing as the PIMS. However, DEPIMS_30_ materials displayed a pronounced loss of scattering intensity at q*, suggesting that 30 mol % DVB did not mechanically reinforce the network sufficiently to fully retain the porosity created by depolymerizing the P­(MMA-*co*-PhthMA) phase. This coincides with previous reports which have demonstrated that high cross-linking densities are required to stabilize mesoporous polymers of many types against pore collapse at high temperatures.^20,25^ DEPIMS materials with DVB content greater than 50 mol % retained strong scattering at q*, which agrees well with the visual appearance of these materials shown via SEM. The increasingly rough texture of the surface of DEPIMS materials at higher DVB content revealed by SEM is supported by Porod analysis of SAXS patterns at high *q* (Figure S14).

Nitrogen sorption isotherms revealed the pronounced influence of cross-link density on the surface area of materials prepared by DEPIMS, reinforcing the link between domain retention and accessible porosity. Brunauer-Emmet-Teller (BET) analysis revealed that surface area increased from 97 to 220 m^2^/g from DEPIMS_30_ to DEPIMS_50_ before plateauing around 280 m^2^/g for DEPIMS_70_ and DEPIMS_100_ (Figure 4C and Table S1). The low surface areas of DEPIMS_30_ are reasonable considering the visual pore collapse observed by SEM and loss of scattering intensity at q* observed via SAXS (Figure 4A,B). Pore sizes were estimated by Barrett–Joyner–Halenda (BJH) modeling of the desorption isotherm, revealing pores of 7 nm for all samples except DEPIMS_100_, which exhibited pores of 5 nm (Figure S15). The incomplete depolymerization observed via TGA-MS could have resulted in residual PMMA coating the pore walls of the DEPIMS_100_ materials, reducing the average pore volume relative to other DEPIMS formulations. Alternatively, the rapid, early cross-linking that roughens the phase interfaces of DEPIMS_100_ could have also reduced the pore size by freezing developing pores in the early stages of phase separation. Importantly, BJH analysis tends to model narrow necks within pores, which explains the discrepancy between the pore size estimation and the pore sizes observed via SEM. Importantly, estimates of pore volume and measured pore volume are comparable, and cumulative pore surface area and volume estimates suggest pores in the 10 nm range, which is in good agreement with the domain spacing determined via SAXS (Figure 4B, Figure S16, and Table S1).

The stability of the DEPIMS materials at high temperatures is remarkable, considering previously etched PIMS lost significant surface area upon heating to moderate temperatures (170 to 140 m^2^/g after 1 h at 100 °C).^20^ We attribute the robust mesopores formed in these networks to additional curing that occurs during fabrication. Indeed, differential scanning calorimetry (DSC) of PIMS_100_ materials heated at 5 °C/min revealed an exothermic curing peak at 120–220 °C, which offers evidence for reactions of residual vinyl groups of DVB (Figure S17). There is no evidence of additional curing occurring when heating the resulting DEPIMS_100_ materials to the same temperature range, suggesting the residual vinyl groups of DVB are consumed during the DEPIMS process.

We reasoned that the large surface areas and well-defined pores exhibited by the DEPIMS materials could make them attractive adsorbents if the mesoporous surface was decorated with appropriate functional groups. As a demonstration, we performed post-polymerization sulfonation of the nanoporous polystyrene monoliths by immersing crushed DEPIMS powders into H_2_SO_4_ at 70 °C for 6 h (Figure 5A). The resulting sulfonated DEPIMS materials (S-DEPIMS) were collected via filtration and washed with deionized water until the filtrate was pH-neutral. FTIR spectroscopy of the resulting materials exhibited signals corresponding to the characteristic O=S=O stretch at 1050–1250 cm^–1^ (Figure 5D). Interestingly, sulfonation was markedly less effective for materials with higher cross-link densities, as evidenced by the dwindling relative intensity of the O=S=O stretch compared to the aromatic C–H out-of-plane bend at 700 cm^–1^.The different extents of sulfonation are not surprising given that the majority of the commercial-grade DVB employed in this study is *para*-substituted, and electrophilic aromatic substitution is disfavored on *para*-substituted aromatic rings. Importantly, sulfonation seems to preserve the porous microstructure of the DEPIMS materials, as evidenced by structure factor peaks in SAXS and a lack of obvious changes to the morphologies of the S-DEPIMS powders in SEM micrographs (Figure 5C and Figures S18 and S19).

**Figure 5:**
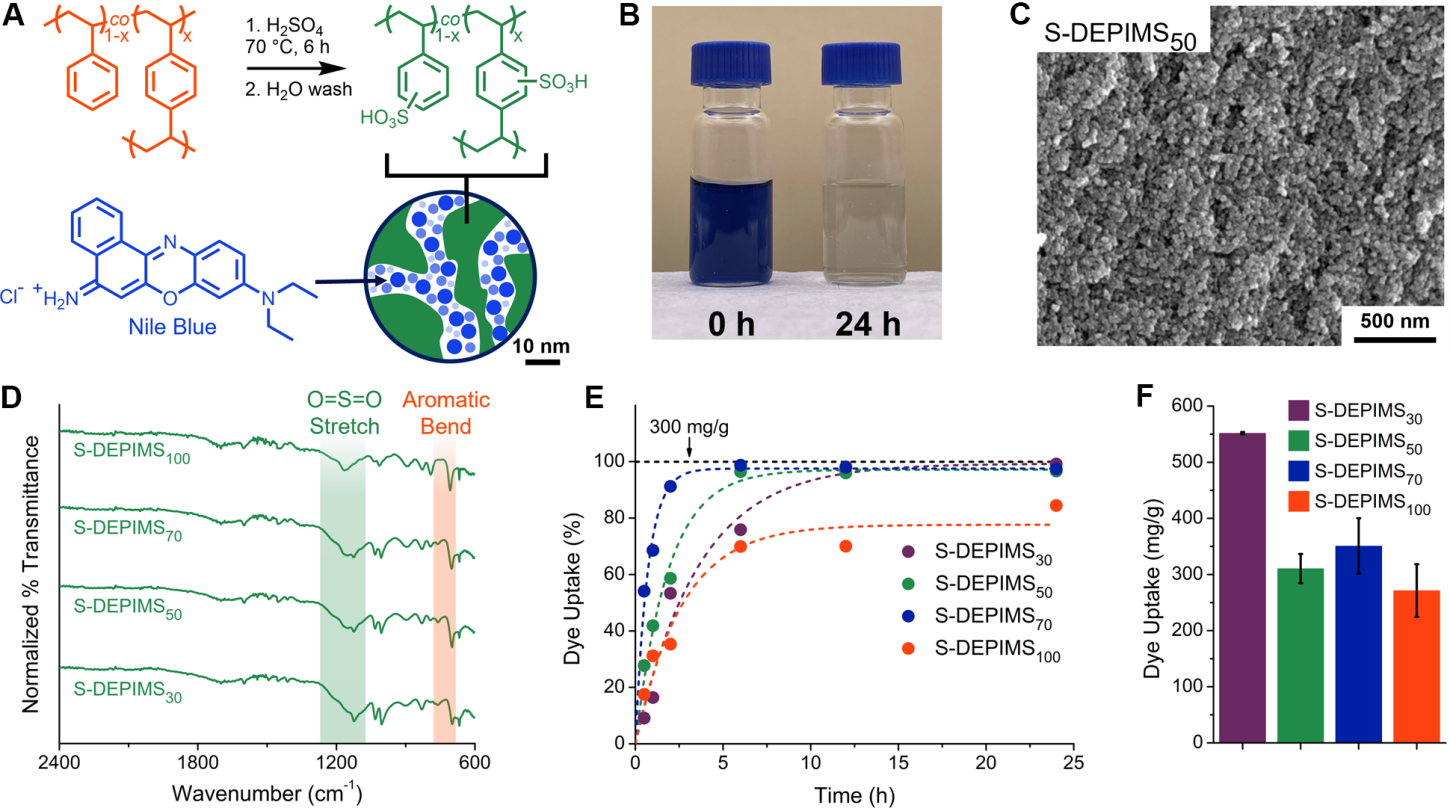
(A) Synthetic approach to sulfonated DEPIMS materials (S-DEPIMS) and schematic demonstrating uptake of cationic dye Nile Blue (NB). (B) Photo of (left) stock solution of 0.1 mg/mL Nile Blue in 0.1 M phosphate buffer at pH = 7 and (right) solution obtained after 15 mL of NB stock solution was stirred with 5 mg of S-DEPIMS_30_ powder for 24 h and filtered. More than 99% of NB has been removed from solution over this time. (C) Scanning electron micrograph of S-DEPIMS_50_ powders which shows the mesoporous architecture is retained after sulfonation. (D) FTIR spectra demonstrating successful sulfonation of DEPIMS. (E) Kinetics of dye uptake upon stirring 15 mL of NB stock solution and 5 mg of S-DEPIMS powder for predetermined times. Dashed lines indicate first-order kinetic fits obtained by least-squares fitting (R^2^ > 0.95) (Table S2). (F) Maximum dye uptake capacity determined by stirring 20 mL of NB stock solution with 2 mg of S-DEPIMS powder for 48 h.

With confidence that S-DEPIMS powders were effectively sulfonated and maintained structural integrity, we evaluated the materials as adsorbents for the cationic model dye, Nile Blue (NB). We evaluated the kinetics of our S-DEPIMS powders by adding 5 mg of S-DEPIMS to 15 mL of an aqueous solution of 0.1 mg/mL NB in 0.1 M phosphate buffer at pH 7 (Figure 5E). Nearly all the cationic NB dye was removed from solution within 24 h for S-DEPIMS containing 30–70% DVB (Figure 5B,E and Figure S20). For samples in this range of cross-link densities, the pseudo first-order rate constant of uptake grew with DVB content, likely due to the increased surface areas obtained from the more highly cross-linked materials facilitating accelerated adsorption of the NB (Table S2). Interestingly, the S-DEPIMS materials prepared with 100% DVB showed the slowest rate of dye uptake and lowest dye uptake at equilibrium despite having surface areas comparable to S-DEPIMS_70_ (Figure 4C and Table S1). We attribute this observation to a lower degree of sulfonation for S-DEPIMS_100_ materials relative to others with more Sty incorporation. These observations indicate that pore-wall chemistry and phase composition jointly govern adsorption kinetics and capacity, suggesting these parameters can be rationally tuned for targeted separations.

To assess the maximum dye uptake capacity, 20 mL of 0.1 mg/mL NB solution was mixed with 2 mg of S-DEPIMS powder, and the mixture was stirred for 48 h. All S-DEPIMS powders displayed high uptake capacities of above 250 mg of NB per gram of S-DEPIMS (Figure 5F and Figures S21 and S22). However, S-DEPIMS_30_ exhibited the highest capacity at 552 ± 1.92 mg/g. This figure exceeds capacity figures from previous PIMS reports (up to 358 mg/g),^28^ emphasizing the versatility of DEPIMS for post-functionalization and application-specific tuning. We reason that the impressive dye uptake capacity of the S-DEPIMS_30_ material is a result of the extensive sulfonation enabled by high Sty content. Future work oriented toward S-DEPIMS adsorbents could benefit from strategies that enhance structural retention at lower DVB content to maximize both the rate and maximum capacity of uptake.

To compare our S-DEPIMS absorbent properties to established materials, we evaluated the dye uptake capacity of a commercially available anionic ion-exchange resin, Amberlite IRC 120 (Figures S23 and 24). Every S-DEPIMS material showed greater dye uptake at 12 h and below, suggesting that the interconnected pores generated by this approach are crucial for facilitating rapid dye uptake. The enhanced uptake kinetics could make S-DEPIMS an attractive option for separations and purification processes where efficient uptake on short time scales is desirable.

To explore scalability, we implemented a one-pot, solvent-free route that produced gram-scale mesoporous materials in under 12 h with recovery of near-pure MMA (Figure 6). This approach integrated polymer synthesis, microphase separation, and selective depolymerization into a single workflow. MMA and PhthMA were copolymerized via RAFT in bulk at 90 °C for 3 h. In that time, the polymerization reached 92% monomer conversion, and the resulting P­(MMA-*co*-PhthMA) macroCTA was 68.0 kDa with a *Đ* = 1.03 (Figure S25). The macroCTA was dissolved at 30 wt % in a 70:30 molar mixture of DVB:Sty and heated to create PIMS (PIMS_B_) materials in the bulk via a one-pot process. PIMS_B_ was depolymerized in bulk in a distillation apparatus (PIMS_BD_) at 290 °C for 30 min (Figure 6A,B and Figure S26). The distillate was primarily MMA, as indicated by ^1^H NMR spectroscopy, and was recovered in good yield (∼65%) (Figure S27). TGA-MS and FTIR spectroscopy revealed that bulk DEPIMS (DEPIMS_B_) displayed less selectivity during the etching process relative to DEPIMS_70_ materials, with a broader depolymerization mass loss window and less efficient MMA removal (Figure 6C and Figure S28). However, the structure, surface area, and dye uptake characteristics were similar to the DEPIMS materials prepared in a stepwise fashion (Figure 6D–F, Figures S29 and S30, and Tables S3 and S4), suggesting the DEPIMS approach can be leveraged for accelerated synthesis of mesoporous materials.

**Figure 6:**
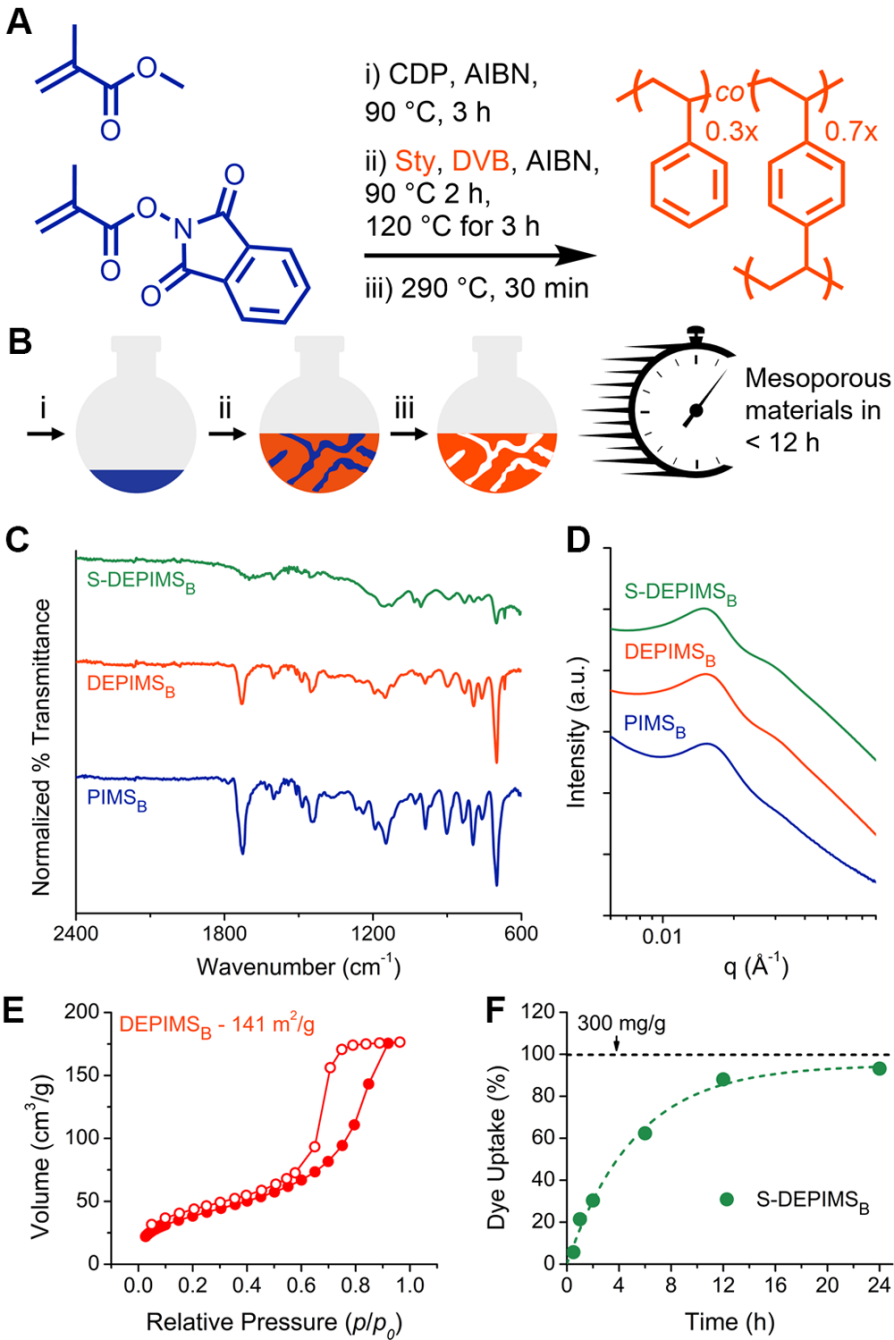
(A) Synthetic approach to one-pot DEPIMS materials (DEPIMS_B_) generated in bulk. (B) Schematic of one-pot DEPIMS process used to generate DEPIMS_B_. (C) FTIR spectra of DEPIMS_B_ materials generated via bulk distillation and S-DEPIMS_B_ obtained via sulfonation of DEPIMS_B_ materials. (D) SAXS patterns from PIMS_B_, DEPIMS_B_, and S-DEPIMS_B_ that indicate structure is retained for each material. Scattering patterns are vertically shifted for clarity. (E) Nitrogen sorption isotherms with BET surface areas suggesting modest surface areas for DEPIMS_B_, which we attribute to inefficient depolymerization (Figure S23). Details of BET surface area fitting can be found in Table S3. Adsorption and desorption isotherms are indicated by filled and unfilled circles, respectively. (F) Kinetics of dye uptake upon stirring 15 mL of NB stock solution and 5 mg of S-DEPIMS_B_ powder for predetermined times. Dashed lines indicate first-order kinetic fits obtained by least-squares fitting (R^2^ > 0.95) (Table S2).

## Conclusion

We have established bulk thermal depolymerization as an effective design strategy for fabricating well-defined mesoporous materials through the development of the DEPIMS process. Unlike conventional polyester hydrolysis used in mesoporous PIMS synthesis, the selective thermal depolymerization of polymethacrylates enables phase separation with fewer synthetic steps and generates pores significantly faster than previous solvent-based etching approaches by using in situ generation of gaseous monomer upon depolymerization as an etching technique. TGA-MS analysis confirmed the high selectivity of DEPIMS for removing MMA while preserving the integrity of the polystyrenic matrix. Comprehensive structural characterization (SEM, SAXS, and nitrogen sorption) demonstrated that sufficiently cross-linked DEPIMS materials retain robust mesoporous architectures and high surface areas (up to 278 m^2^/g) after -depolymerization. Subsequent sulfonation yielded materials with exceptional dye adsorption performance, including the highest reported uptake for mesoporous PIMS (552 mg/g) and rapid uptake kinetics. We further demonstrate that DEPIMS materials can be synthesized via a one-pot, solvent-free process within 12 h, offering efficient MMA recovery while maintaining key structural and functional features. These results position DEPIMS as a conceptually distinct and versatile strategy for engineering mesoporous materials with broad implications for energy, environmental remediation, and sustainable manufacturing. By enabling thermally triggered pore generation without solvents or harsh conditions, DEPIMS opens a new design space for functional nanomaterials that unites sustainability, structural control, and on-demand reactivity.

## Supplementary Material



## Notes

Materials, instrumentation, procedures, supplementary figures and tables, and references (PDF)
