# Supplementary material for: Depolymerization as a Design Strategy: Depolymerization Etching of Polymerization-Induced Microphase Separations
Source: ACS Cent Sci. 2025 Oct 29;11(12):2366–74. doi: 10.1021/acscentsci.5c01313 (PMC12746147; doi:10.1021/acscentsci.5c01313)
Supplement: Supplementary file 1 [file oc5c01313_si_001.pdf]

Supplemental Information for

Depolymerization as a Design Strategy: Depolymerization Etching of Polymerization-Induced Microphase Separations

Kaden C. Stevens,[1,*] Megan E. Lott,[1] Kiana A. Treaster,[1] Robert M. O'Dea,[2] Adarsh Suresh,[3] Cabell B. Eades,[1] Victoria L. Thompson,[1] Jared I. Bowman,[1] James B. Young,[1] Austin M. Evans,[1] Stuart J. Rowan,[3] Thomas H. Epps, III,[2] Brent S. Sumerlin[1,*]

*Correspondence: sumerlin@chem.ufl.edu, kaden.stevens@usm.edu

[1]George and Josephine Butler Polymer Research Laboratory, Center for Macromolecular Science & Engineering, Department of Chemistry, University of Florida, Gainesville, FL 32611, USA

[2]Department of Chemical and Biomolecular Engineering, University of Delaware, Newark, DE, 19716, USA, Center for Plastics Innovation (CPI), University of Delaware, Newark, DE 19716, USA, Center for Research in Soft matter and Polymers (CRiSP), University of Delaware, Newark, DE 19716, USA

[3]Prtizker School of Molecular Engineering, The University of Chicago, Chicago 60637, IL, USA

Supporting Information:

S1. Materials

S2. Instrumentation

S3. Procedures

S4. Supplementary Figures and Tables

S5. References

S1. Materials

All chemicals were used as received unless otherwise noted. Methyl methacrylate (MMA, Thermo Scientific, 99%), Styrene (Sty, Fischer Chemical, 99.9%), divinyl benzene (DVB, Sigma-Aldrich, 80%) were passed through a plug of basic alumina to remove inhibitor immediately before use. Dichloromethane (DCM), acetone, dimethyl sulfoxide (DMSO, ACS grade), and *N*,*N*-dimethylformamide (DMF, ACS grade) were purchased from ThermoFisher Scientific. Deionized water was purchased from Fischer Chemicals (HPLC grade). Nile Blue chloride was purchased from Sigma-Aldrich (85%). *N*-Hydroxyphthalimide was purchased from TCI Chemicals. Methacryloyl chloride was purchased from Sigma-Aldrich. Deuterated chloroform ($CDCl_3$, 99.8% with 0.05% v/v TMS) was purchased from Cambridge Isotope Laboratories. 2,2'-Azobis(isobutyronitrile) (AIBN) was purchased from Fujifilm Wako Chemicals and recrystallized in ethanol before use. 4-Cyano-4-[(dodecylsulfanylthiocarbonyl)sulfanyl]pentanoic acid (CDP) and phthalimide methacrylate (PhthMA) were synthesized according to previously reported literature procedures.[1,2]

S2. Instrumentation

NMR Spectroscopy. ^{1}H NMR spectra were recorded using a Bruker Ascend 400 MHz spectrometer. Chemical shifts (δ) are given in parts per million (ppm) relative to TMS and referenced to residual protonated solvent purchased from Cambridge Isotope Laboratories, Inc. ($CDCl_3$: δ^1H 7.26 ppm).

Size-Exclusion Chromatography (SEC). Organic-phase SEC was performed using *N*,*N*-dimethylacetamide (DMAc) salted with 50 mM LiCl and held at at 50 °C with a flow rate of 1.0 mL/min using an Agilent SEC system equipped with a Viscogel I-series 5 μm guard + two ViscoGel I-series G3078 mixed bed columns with molecular weight ranges of 0−20 and 0−10,000 kg/mol. Refractive index and light scattering detection were performed with a Wyatt Optilab T-rEX refractive index detector operating at 658 nm and a Wyatt miniDAWN TREOS light scattering detector operating at 690 nm, respectively. Absolute molecular weights and molecular weight distributions were calculated using the Wyatt ASTRA software and d*n*/d*c* values obtained from 100% mass recovery methods.

Thermogravimetric Analysis (TGA). TGA experiments using a TA 5500 equipped with an autosampler using a 100 µL platinum pan. Each sample was run after precipitation and vigorous drying under vacuum. Ramp experiments were heated at 10 °C/min unless otherwise stated from room temperature to 500 °C under nitrogen flow (25 mL/min). All isothermal experiments were conducted by heating at 50 °C/min to 290 °C and holding for 120 min. Low-temperature TGA experiments were recorded using TA's Thermal Advantage for Q Series software. Mass loss is correlated with the extent depolymerization mentioned in the text.

Tandem Mass Spectrometry. A TA 5500 series TGA was equipped with an MKS DMS Series ll mass spectrometer to analyze ion fragments from polymer samples. The instrument utilizes a 1-300 amu quadrupole mass filter with a closed ion source. Bar chart experiments were used to scan all ion fragments from polymer samples, with peak jump mode used to detect discrete ions for

tracking the generation of identifiable ions. Data were processed using ProcessEye software and exported as text files to Origin.

Differential Scanning Calorimetry (DSC). DSC experiments were conducted on a TA Q2500 DSC (TA Instruments, New Castle, DE) equipped with an autosampler and refrigerated cooling system 90 using aluminum hermetic sealed pans. Ramp experiments were performed by heating and cooling DSC pans under nitrogen (25 mL/min) at 5 °C/min. All DSC experiments were recorded using the Thermal Advantage for Q Series software from TA.

UV-Vis Spectroscopy UV-Vis spectra of dye-containing solutions was obtained with an Agilent Cary 5000 ultraviolet-visible spectrometer from 200-800 nm. The spectra in solution were obtained using disposable glass cuvettes with a 1 cm path length.

FTIR Spectroscopy. Infrared spectra were acquired on a PerkinElmer Spectrum One FTIR spectrometer equipped with a PIKE MIRacle single reflection 3 ATR accessory containing a diamond crystal sample plate. Spectra were processed using PerkinElmer Spectrum 10 software.

Small-angle X-ray Scattering Small angle X-ray scattering (SAXS) was conducted on powder samples using a Xenocs Xeuss 2.0 equipped with a Cu source (λ = 0.154 nm) and a Pilatus 300K pixel array detector (pixel size 172 μm). Samples were loaded into a multi-sample holder and sealed with Kapton tape before being placed in the sample chamber. The chamber then was evacuated prior to data collection. The sample-to-detector distance was 1,500 mm, and the acquisition time was 3 h for each sample. 2D data was azimuthally integrated to generate 1D plots using the Foxtrot software package (version 3.5.10).

Scanning Electron Microscopy Scanning electron microscopy (SEM) was conducted with Carl Zeiss Merlin high-resolution Field Emission Scanning Electron Microscope (FE-SEM). The SEM was operated under the In-Lens mode with an accelerating voltage of 10 kV. The samples were placed on copper tape stuck onto stubs and sputter-coated with a thin layer (~4 nm) of Pt/Pd alloy to improve electron conductivity.

Nitrogen Porosimetery Nitrogen Sorption Isotherms. Sorption isotherm measurements were collected on a Micromimetics ASAP 2020 Plus Accelerated Surface Area and Porosity analyzer. Approximately 100-200 mg of polymer sample was transferred to a dried analysis tube and sealed with a Transeal cap. The sample was heated to 40 °C at a rate of 1°C/min and evacuated at 40°C for 20 min. Then, the sample was heated to 100°C at a rate of 1°C/min and evacuated at 100 °C until the outgas rate was ≤0.3 μmHg min^{-1}. The tube was reweighed to determine the mass of the activated sample and subsequently transferred to the analysis port to begin analysis. N_2 was used for all adsorption measurements. N_2 isotherms were generated by incremental exposure to nitrogen up to 760 mmHg (1 atm) in a liquid nitrogen (77 K) bath. Brunauer-Emmett- Teller (BET) surface areas were calculated from the linear region of the N2 isotherm.

S3. Procedures

MacroCTA Synthesis

The poly(MMA-*co*-PhthMA) macroCTA was synthesized by adding MMA (8.00 g, 425 equiv), PhthMA (1.08 g, 25.0 equiv), CDP (75.9 mg, 1.00 equiv), AIBN (3.08 mg, 0.100 equiv), and dioxane (42.3 mL) to a round-bottom flask along with trioxane (0.2 g) as an internal standard. The mixture was sparged under argon for 20 min before being placed in a preheated oil bath (70 °C) for 25 h. The resulting polymer was precipitated into cold methanol three times and thoroughly dried under vacuum before use ($M_{n,SEC}$ – 40.5 kg/mol, $Đ$ = 1.03). PhthMA content was determined to be 8 mol% via ^{1}H NMR spectroscopy.

Stepwise DEPIMS Procedure

Depolymerization etching of polymerization-induced microphase separations (DEPIMS) was performed in a stepwise manner via the following general procedure. To generate $PIMS_{30}$, macroCTA (300 mg, 30 wt%), Sty (503 µL, 0.7 equiv), and DVB (266.2 µL, 0.3 equiv) were added to a 2 dram vial and sparged for 5 min before being placed into a preheated oil bath (120 °C) for 5 h. The resulting $PIMS_{30}$ materials were broken into pieces, which were then heated to 290 °C for 15 min via TGA to generate $DEPIMS_{30}$ materials. All DEPIMS materials went from yellow before depolymerization to brown after depolymerization, likely due to degradation of the CTA at elevated temperatures. Additionally, cracks developed in the DEPIMS materials after thermal treatment.

Bulk DEPIMS Procedure

Bulk DEPIMS materials were generated by mixing MMA (2.00 g, 554 equiv) PhthMA (217 mg, 27.7 equiv), CDP (14.5 mg, 1.00 equiv), and AIBN (1.08 mg, 0.200 equiv) in a round-bottom flask and sparging this mixture for 10 min before being placed in a preheated oil bath at 90 °C for 3 h. Upon cooling, 53 mg of the poly(MMA-*co*-PhthMA) was extracted and analyzed to reveal the polymerization reached 92% monomer conversion as determined by ^{1}H NMR spectroscopy. The resulting polymer was analyzed by SEC-MALS ($M_{n,SEC}$ = 68.1 kDa, $Đ$ = 1.03). This macroCTA was dissolved at 30 wt% in DVB (3.30 mL, 0.300 equiv) and Sty (1.14 mL, 0.700 equiv) and stirred for 2 h at 55 °C using a preheated water bath. After dissolution, this mixture was sparged with argon for 15 min and placed into a preheated oil bath at 90 °C for 2 h to cure below the boiling point of the residual PMMA before being heated to 120 °C for 3 h. 277 mg of the $PIMS_B$ material was removed to allow for characterization. The round-bottom flask containing the remaining $PIMS_B$ material was placed into an aluminum heating block and equipped with a condenser and a collection flask cooled to -78 °C via a dry ice-acetone bath. The round-bottom flask was then heated to 290 °C for 30 min and occasionally placed under negative pressure via light vacuum to collect the distillate (1.25 g recovered, which is approximately 66.8% of available MMA) and generate $DEPIMS_B$ materials (4.47 g recovered). The recovered $DEPIMS_B$ materials were used without any further purification.

PIMS Sulfonation Procedure

Sulfonation DEPIMS materials were fabricated by wetting 100 mg of DEPIMS material with ethanol for 2 h at room temperature. Then, the DEPIMS material was mixed with 50 mL of concentrated sulfuric acid in a pressure vessel and heated to 70 °C for 6 h. Finally, the S-DEPIMS materials were cooled to room temperature, filtered and washed until the eluent became neutral.

Dye Uptake Experiments

Nile Blue (NB) was used as a model cationic pollutant. Stock solutions of NB (0.1 mg/mL) in aqueous phosphate buffer (100 mmol, pH = 7) were prepared to study NB uptake by S-DEPIMS. For kinetics experiments, NB stock solution (15 mL) was mixed with S-DEPIMS (5 mg) and stirred at 800 rpm. At pre-determined intervals, the mixture was filtered with 450 µm PTFE filters to remove the S-DEPIMS. To determine uptake capacity, a NB stock solution (20 mL) was mixed with S-DEPIMS (2 mg) and stirred at 800 rpm for 48 h before the mixture was filtered with 450 µm PTFE filters to remove the S-DEPIMS. The remaining dye content in solution was determined via UV-Vis spectroscopy.

S4. Supplemental Figures and Tables

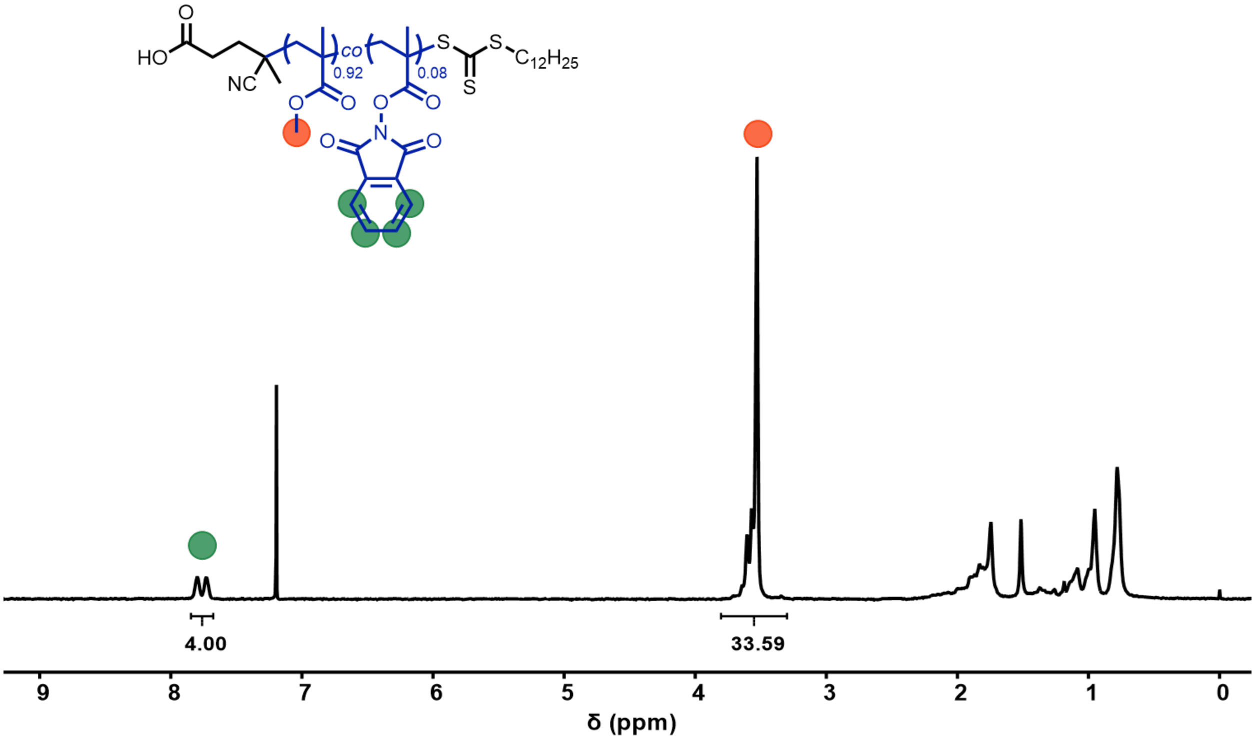

Figure S1. ^{1}H NMR spectroscopy of P(MMA$_{0.92}$-*co*-PhthMA$_{0.08}$) with regions corresponding to the phthalimide aromatic protons and methyl protons integrated to calculate PhthMA incorporation.

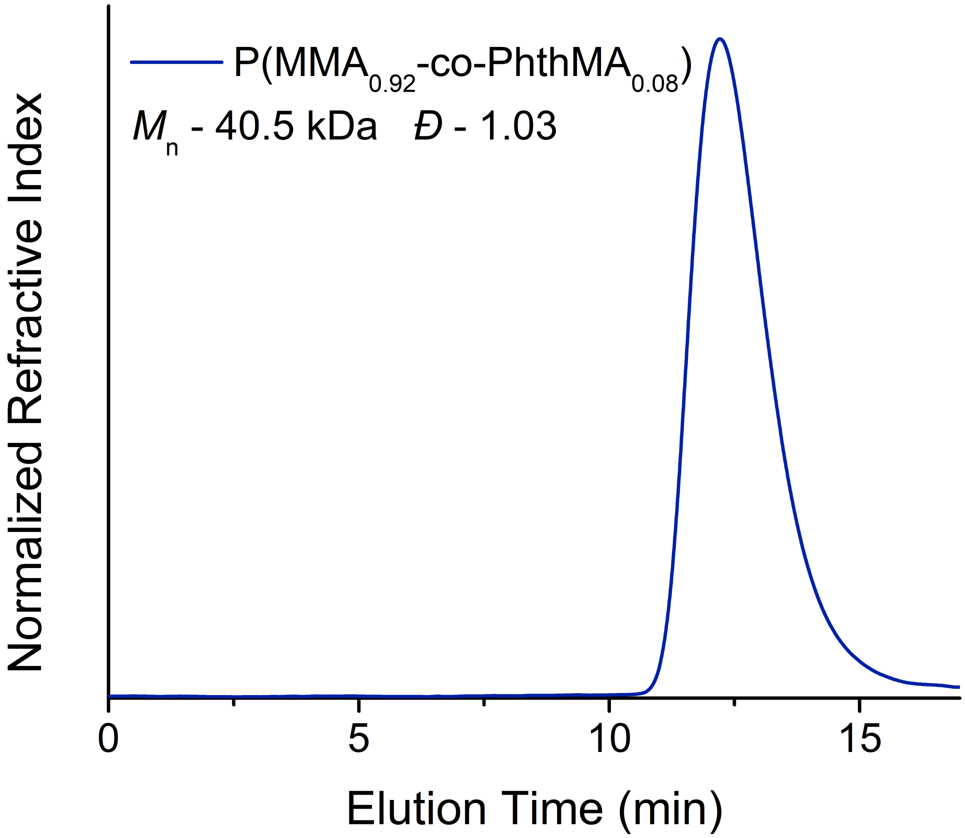

Figure S2. SEC-MALS of P($MMA_{0.92}$-*co*-$PhthMA_{0.08}$).

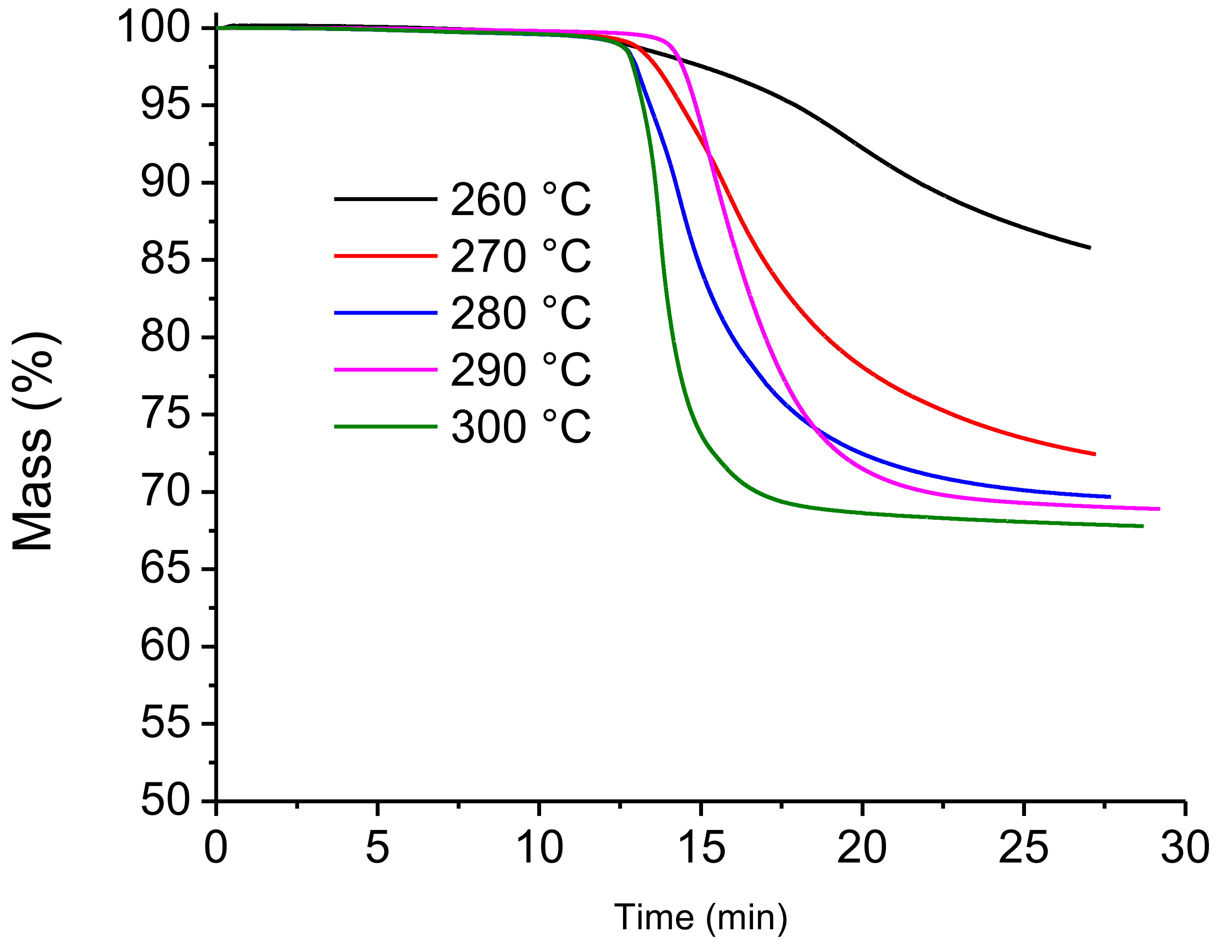

Figure S3. TGA traces for isothermal 15-min holds on $PIMS_{50}$ materials at a range of temperatures. 290 °C was chosen as the mass loss plateaus within the 15-min timeframe without etching more mass than can be attributed to the sacrificial macroCTA.

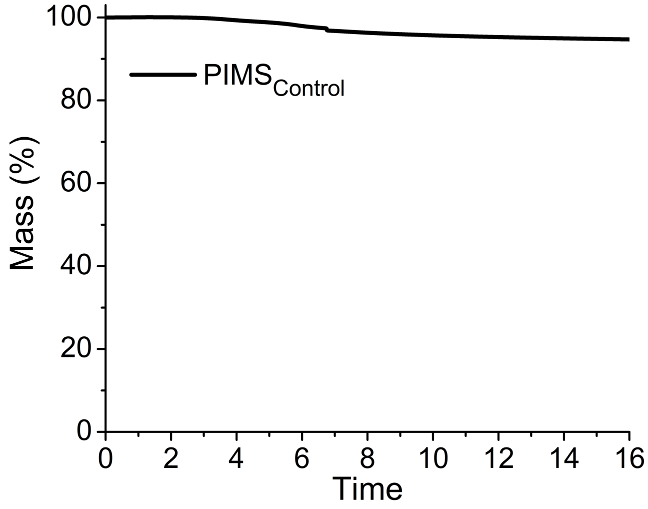

Figure S4. TGA isothermal holds at 290 °C for 15 minutes of PIMS generated using PMMA homopolymer. These samples lose approximately 5% of their original mass.

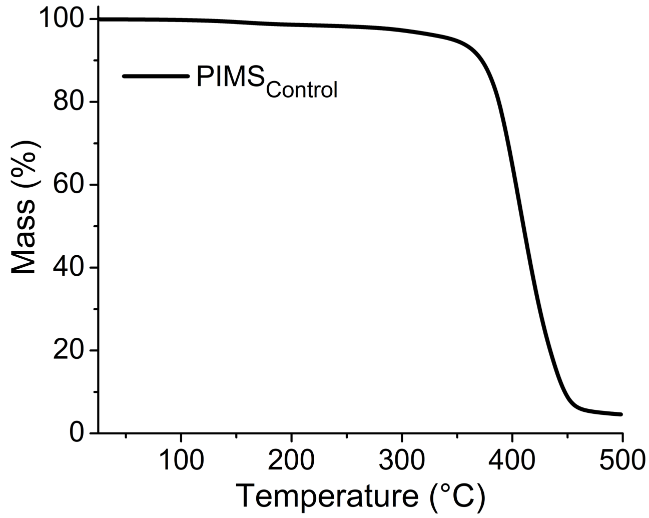

Figure S5. TGA temperature sweeps of PIMS generated using PMMA homopolymer. These sweeps lack the pronounced 30% mass loss between 250–300 °C that occurs when PIMS are fabricated using P(MMA-*co*-PhthMA).

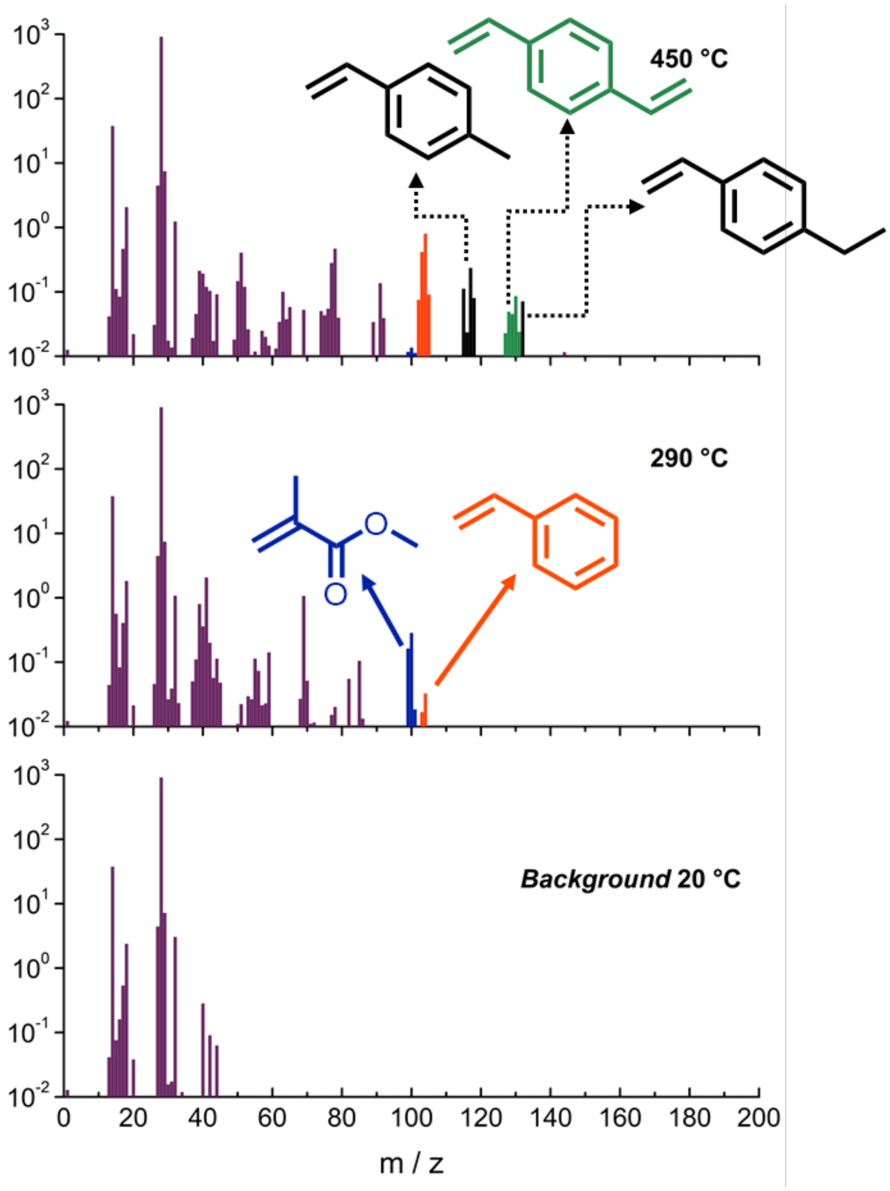

Figure S6. Mass spectrometry analysis of $PIMS_{50}$ materials at 20, 290, and 490 °C with masses corresponding to off-gassed products of interest highlighted. At 290 °C, MMA and Sty are the major products, whereas at 490 °C, mass fragments corresponding to DVB and DVB fragmentation products and impurities are observed.

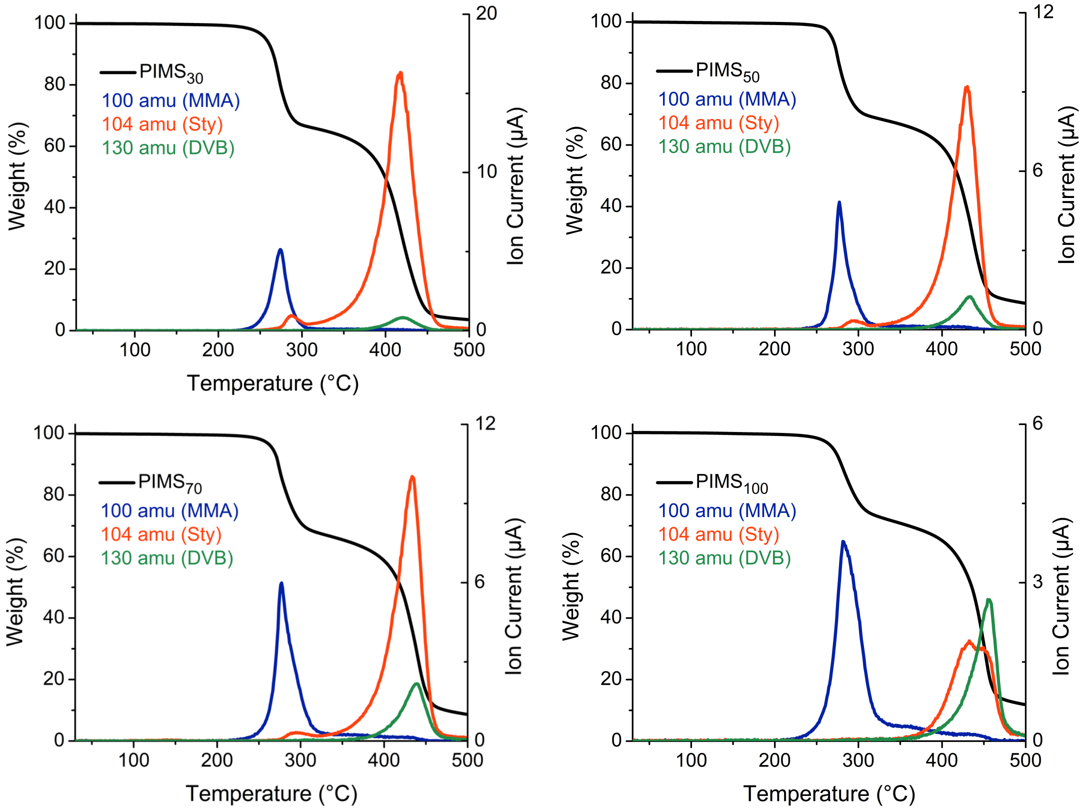

Figure S7. TGA-MS analysis of PIMS materials of varying crosslink density taken to 500 °C at 5 °C/min. MMA, Sty, and DVB are indicated by the blue, orange, and green lines, respectively. As crosslinking density increases, the fraction of off-gassed products in the region from 250 °C to 300 °C shifts towards MMA. At the same time, more MMA is released in the above 400 °C as DVB content increases.

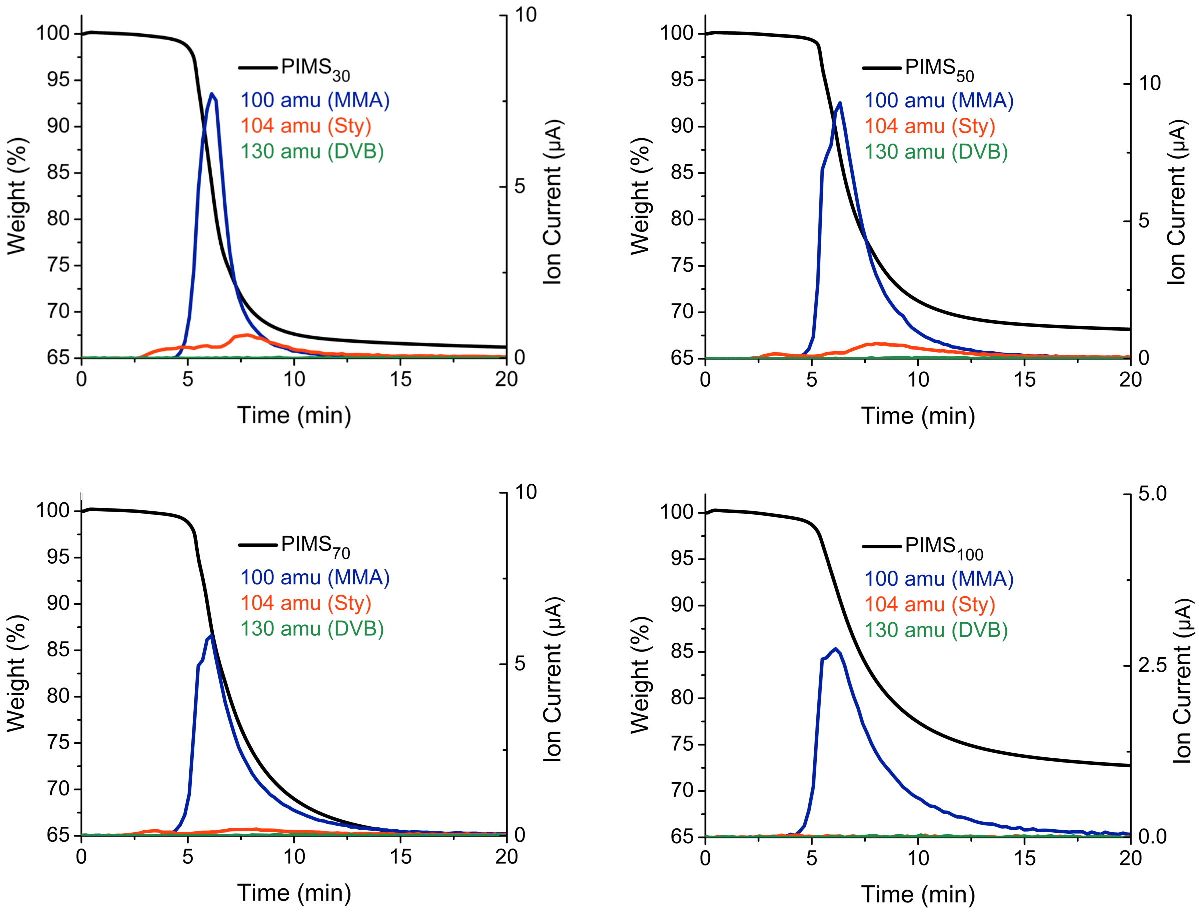

Figure S8. TGA-MS analysis of PIMS materials of varying crosslink density ramped to 290 °C at 20 °C/min and then held at 290 °C for 15 min. MMA, Sty, and DVB are indicated by the blue, orange, and green lines, respectively. As crosslinking density increases, the time necessary for ion current corresponding to MMA to reach the baseline increased. Simultaneously, less Sty was evolved during the isothermal holds as MMA content was increased. DVB extrusion was undetectably low in all experiments.

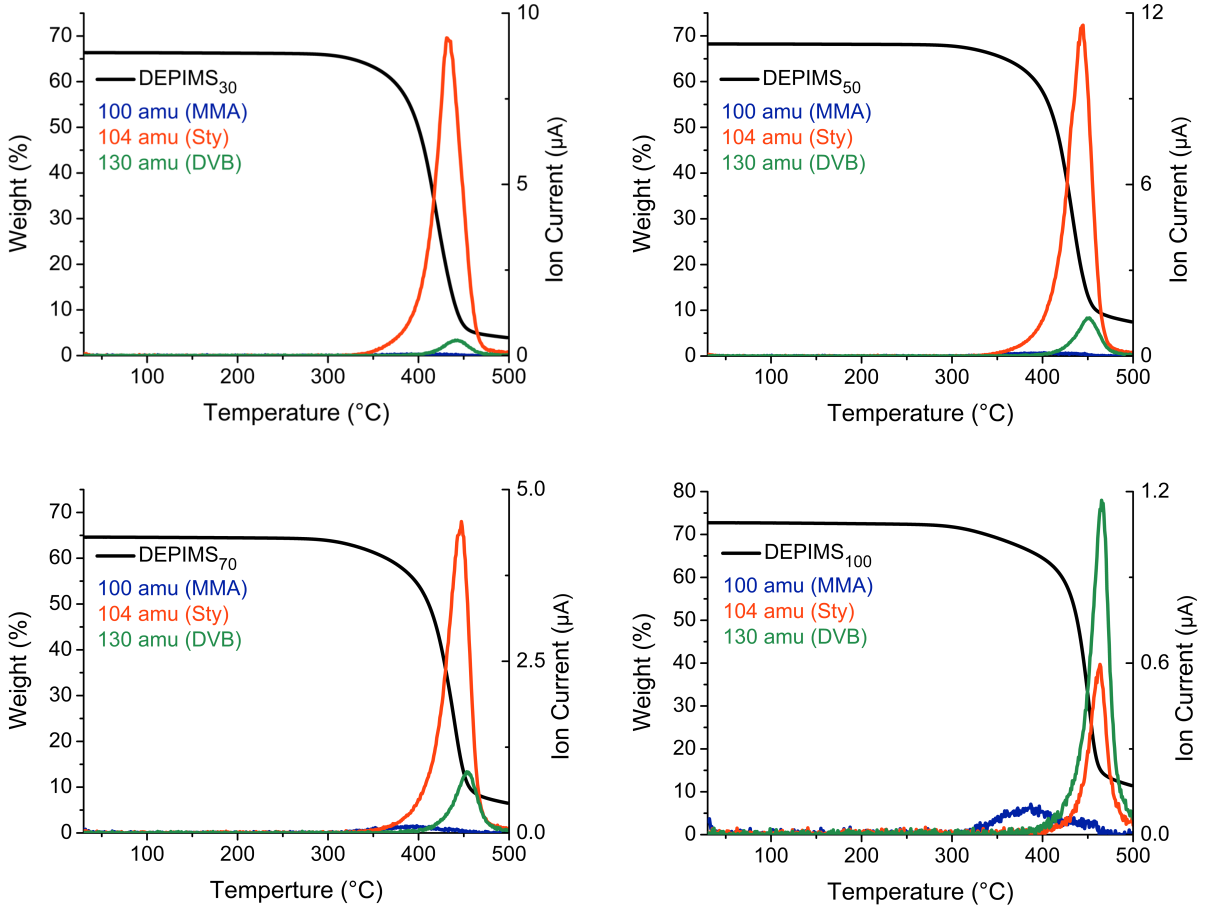

Figure S9. TGA-MS analysis of PIMS materials of varying crosslink density ramped to 500 °C at 5 °C/min. MMA, Sty, and DVB are indicated by the blue, orange, and green lines, respectively. As crosslinking density increases, the time necessary for ion current corresponding to MMA to reach the baseline increased. Simultaneously, less Sty was evolved during the isothermal holds as MMA content was increased. No DVB extrusion was detectable in these experiments.

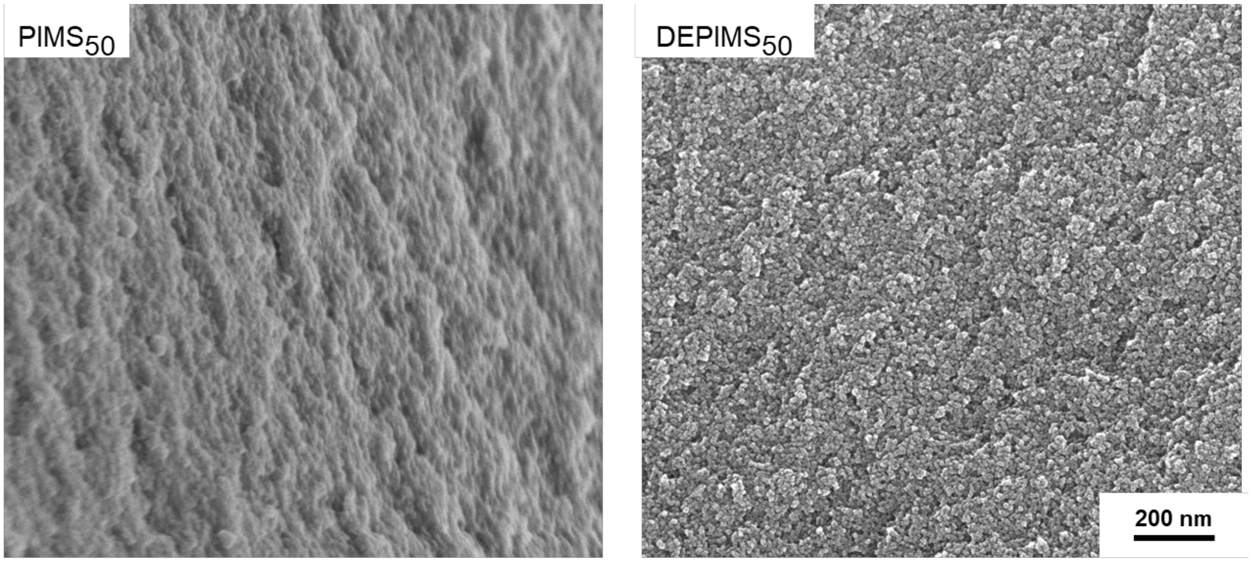

Figure S10. Scanning electron micrographs of $PIMS_{50}$ and $DEPIMS_{50}$ materials demonstrating the absence of porous voids prior to depolymerization and the presence of mesoporous voids after depolymerization.

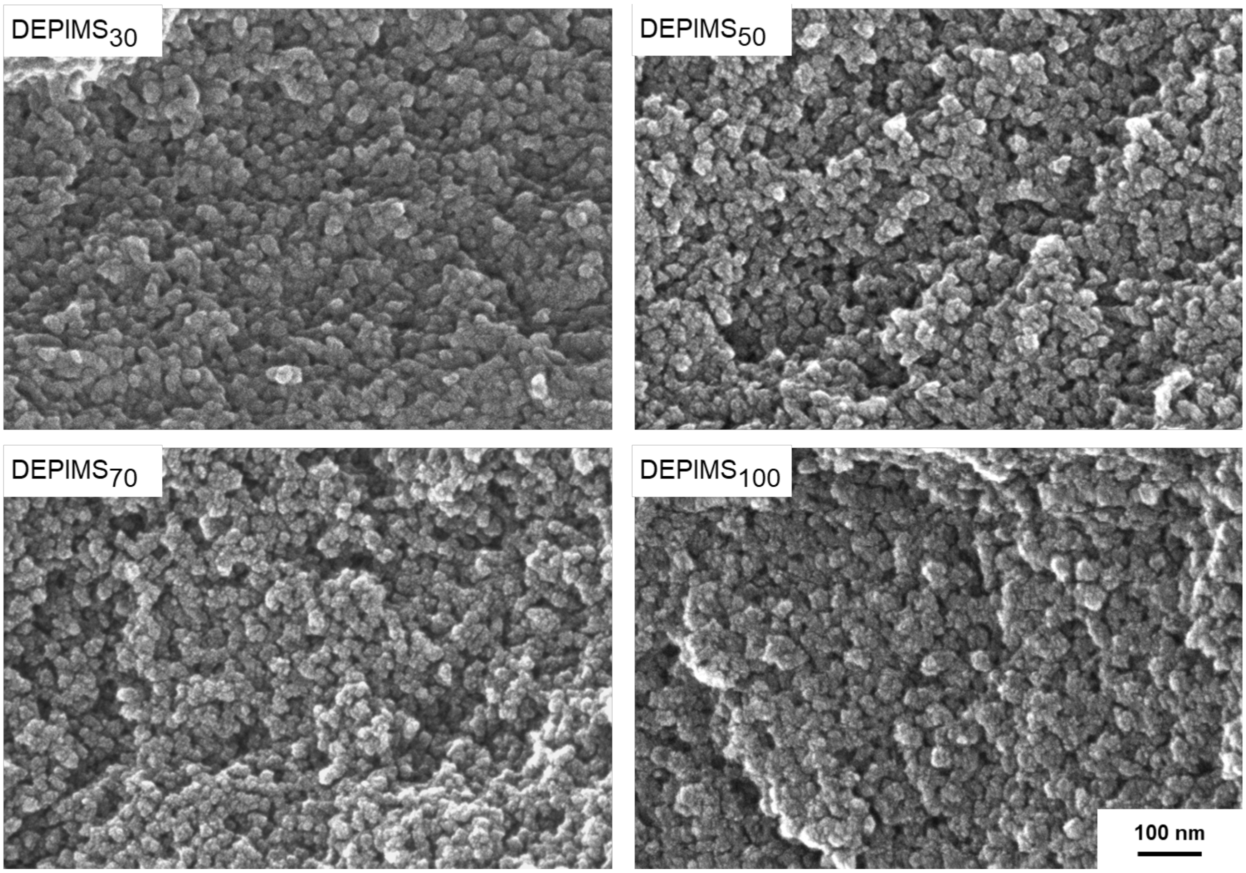

Figure S11. Scanning electron micrographs of DEPIMS materials at varying crosslink densities. The $DEPIMS_{30}$ materials are noticeably damaged by the DEPIMS process.

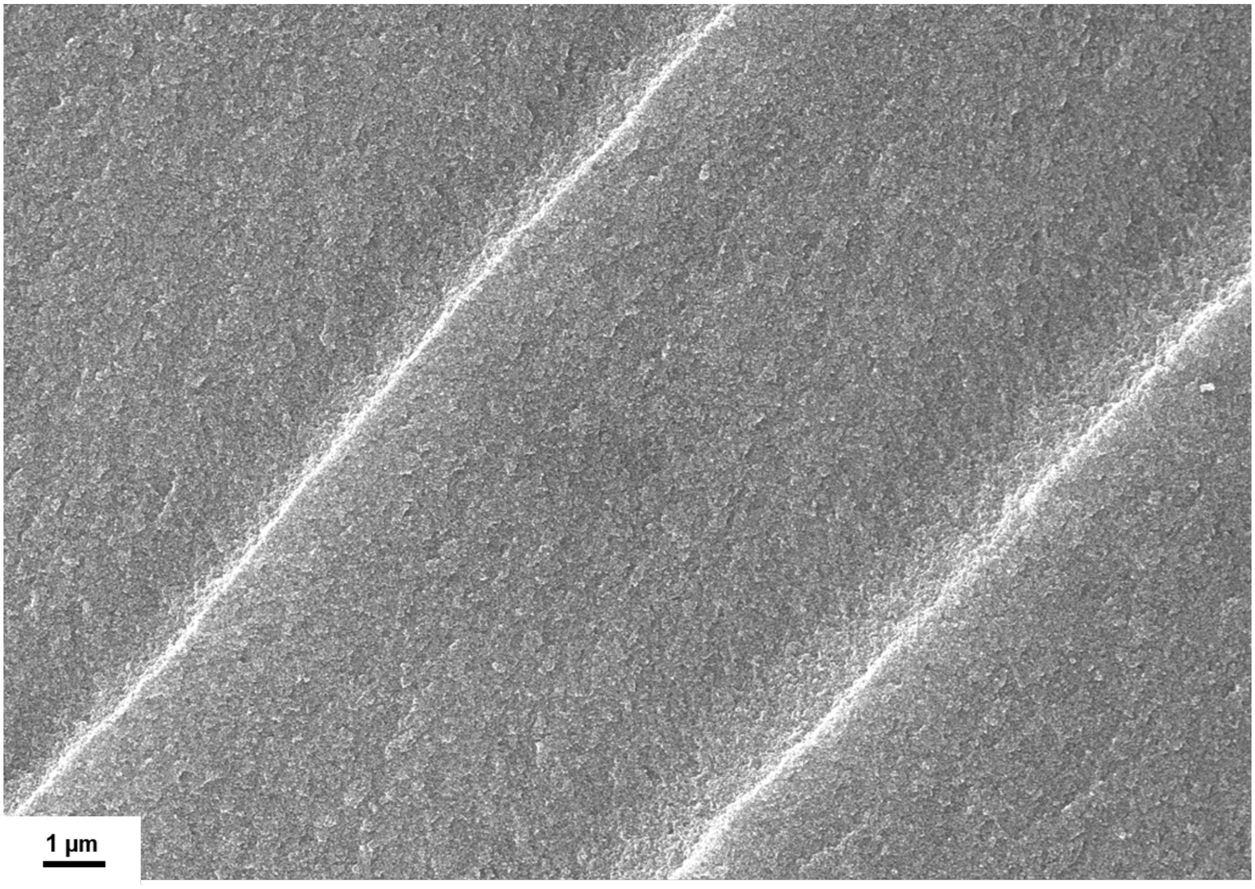

Figure S12. Scanning electron micrograph of $DEPIMS_{70}$ materials demonstrating the uniformity of the mesoporous architecture obtained by the DEPIMS process.

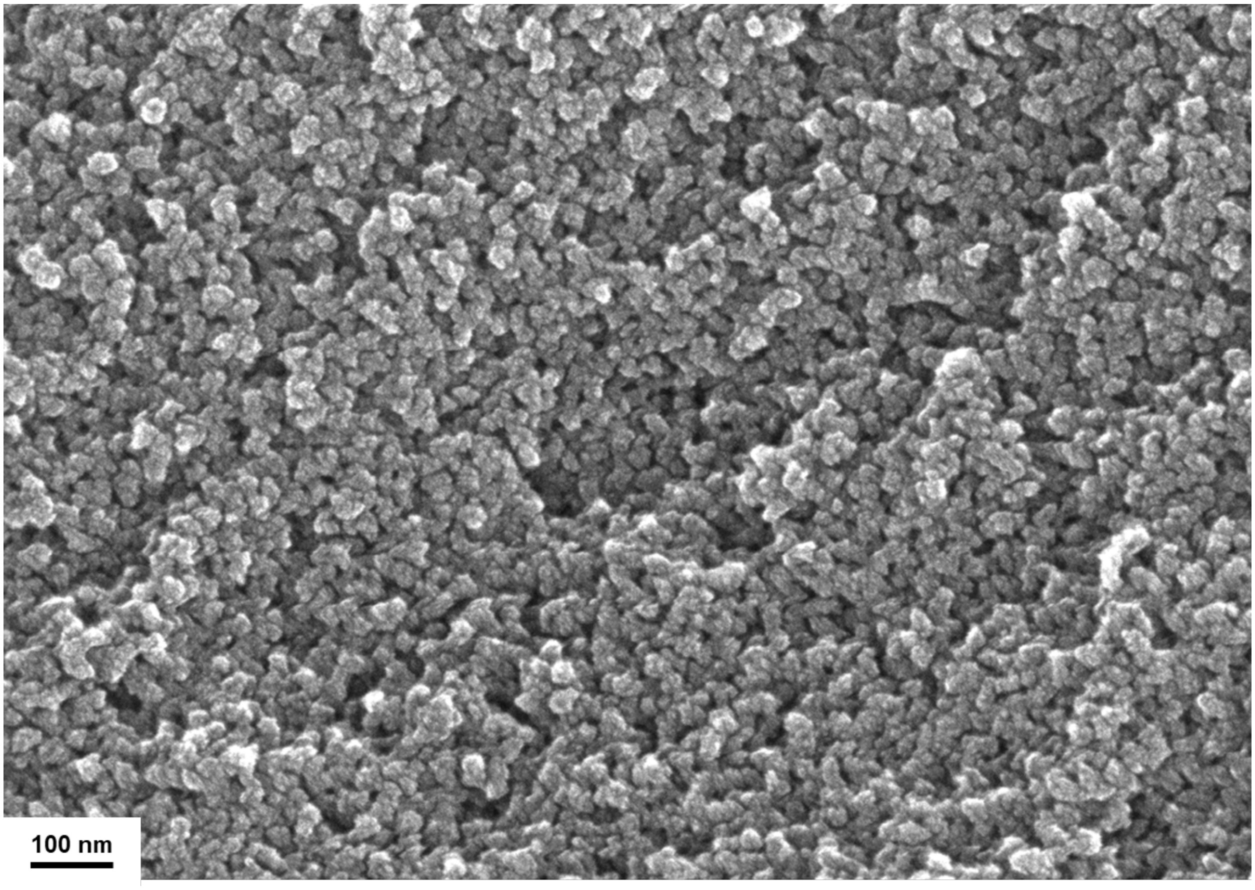

Figure S13. Scanning electron micrograph of $DEPIMS_{70}$ taken by zooming in on a region of DEPIMS material shown in Figure S10, demonstrating the skeletal mesoporous structure remaining after depolymerization of the sacrificial macroCTA.

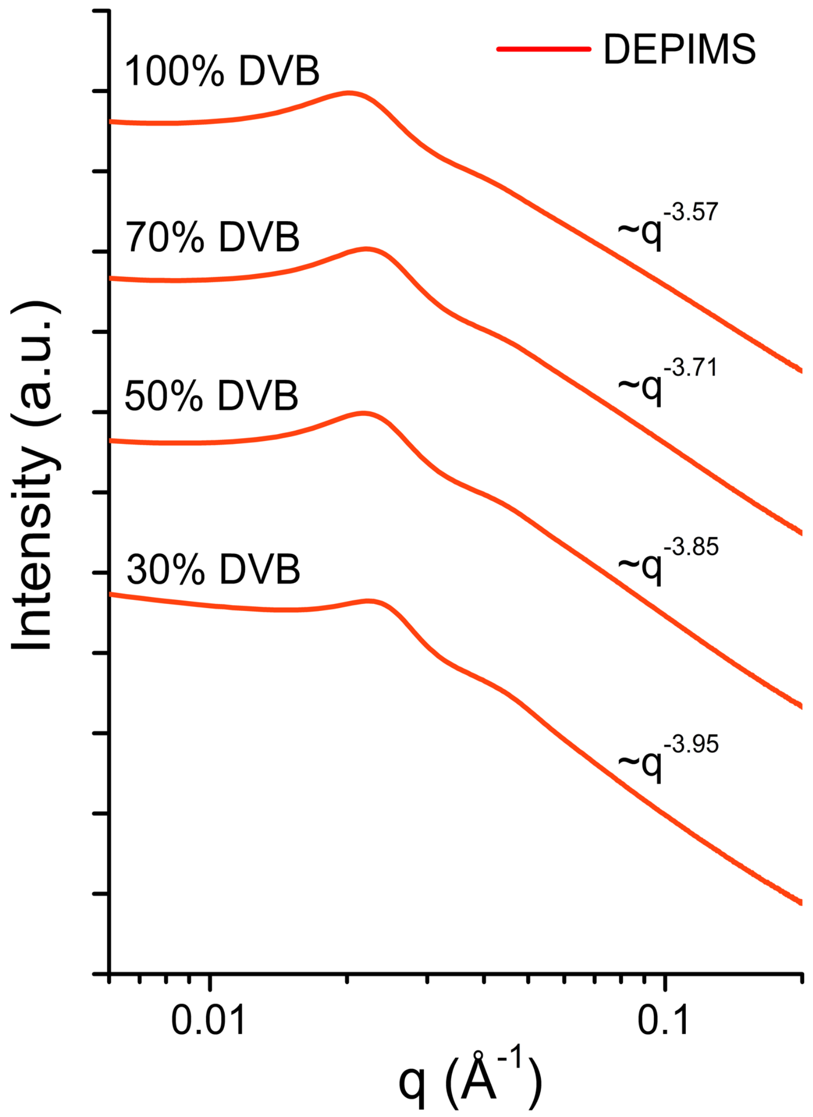

Figure S14. Porod analysis of high q SAXS patterns. In regions where (I(q)) scales with q^{-P}, the Porod exponent (P) can describe the fractal dimensionality of surfaces.[3] Values of P from 3 to 4 result from surface fractals, with 4 indicating a smooth surface and 3 indicating rougher surfaces. P can be directly related to the fractal dimensionality of the surface (D_s) through the relationship $P = 6 - D_s$. As seen above, the Porod exponent decreases from 3.95 for $DEPIMS_{30}$ to 3.57 for $DEPIMS_{100}$, which supports the visual appearance of more diffuse interfaces in the DEPIMS as crosslink density is increased (Figure 4). This has been seen before as a function of increasing crosslinker content and is attributed to increased crosslinking of multifunctional monomers early in the phase separation process leading to rough surfaces between the two phases.

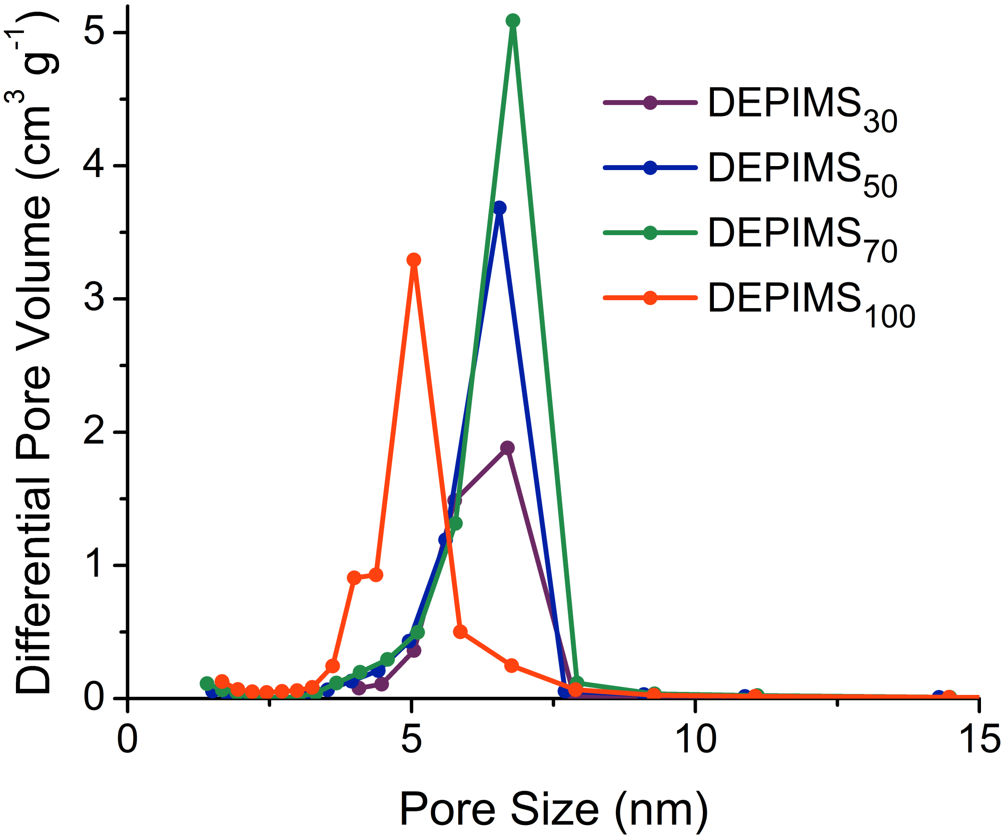

Figure S15. Pore sizes of DEPIMS materials estimated by BJH analysis of the desorption branch of nitrogen porosimetry taken at 77 K.

Table S1. Summary of DEPIMA material analysis obtained via nitrogen porosimetry at 77 K. Estimated from p/p_o of 0.05 to 0.035. Goodness of fit indicated by R^2.*

Sample	BET Surface Area (m^2/g)	R^2	Micropore Area (m^2/g)	Total Volume (cm^3/g)
S-DEPIMS$_{30}$	95.5	0.9998	11	0.17
S-DEPIMS$_{50}$	219	0.9999	8	0.36
S-DEPIMS$_{70}$	275	0.9999	1	0.44
S-DEPIMS$_{100}$	278	0.9999	12	0.34

*Total surface area is estimated to be 0.33 cm^{3}g^{-1} assuming a density for P(MMA-*co*-PhthMA) of 1.2 g/cm^3 and a density for P(St-*co*-DVB) of 1.05 g/cm^3.

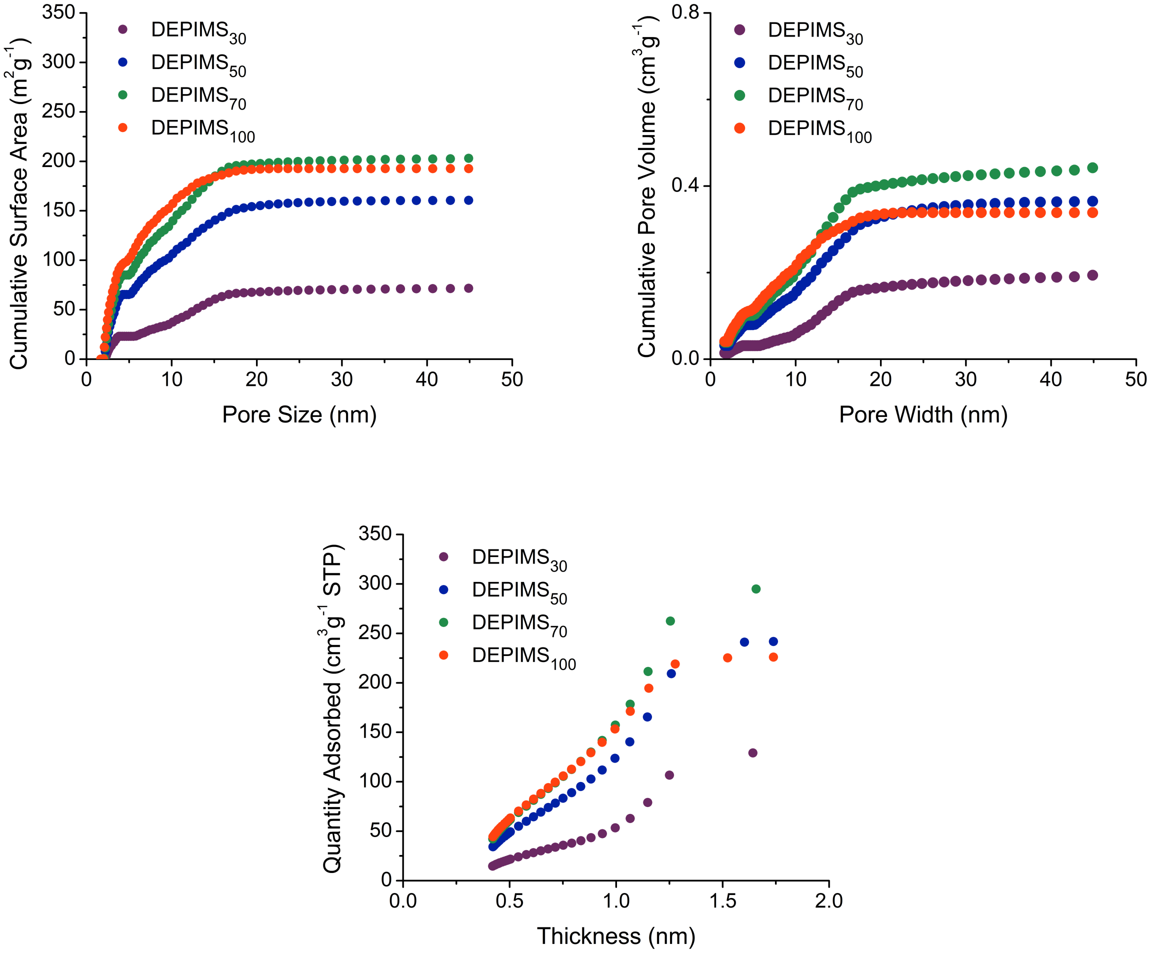

Figure S16. (Top Left) Cumulative surface area, (Top Right) cumulative pore volume and (Bottom) t-plot for DEPIMS materials obtained via N_2 porosimetery.

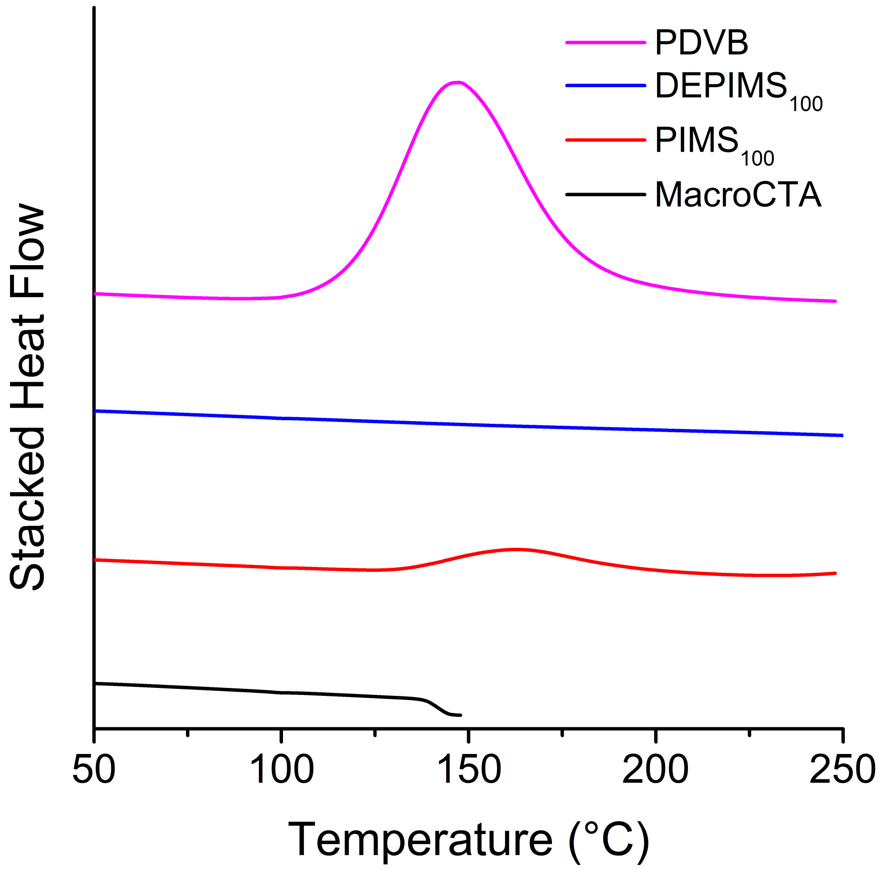

Figure S17. DSC of divinylbenzene polymerized under an inert atmosphere for 2 h at 120 °C (PDVB) to leave residual double bonds for which curing could be easily observed via DSC, as well as $DEPIMS_{100}$ materials, $PIMS_{100}$ materials, and the P(MMA-*co*-PhthMA) macroCTA. The presence of curing peaks in the PDVB and $PIMS_{100}$ materials and absence of curing peaks in the $DEPIMS_{100}$ materials suggest all remaining residual double bonds were cured during the DEPIMS process, which rigidified the network and helps rationalize the resilience of DEPIMS structures towards elevated temperatures.

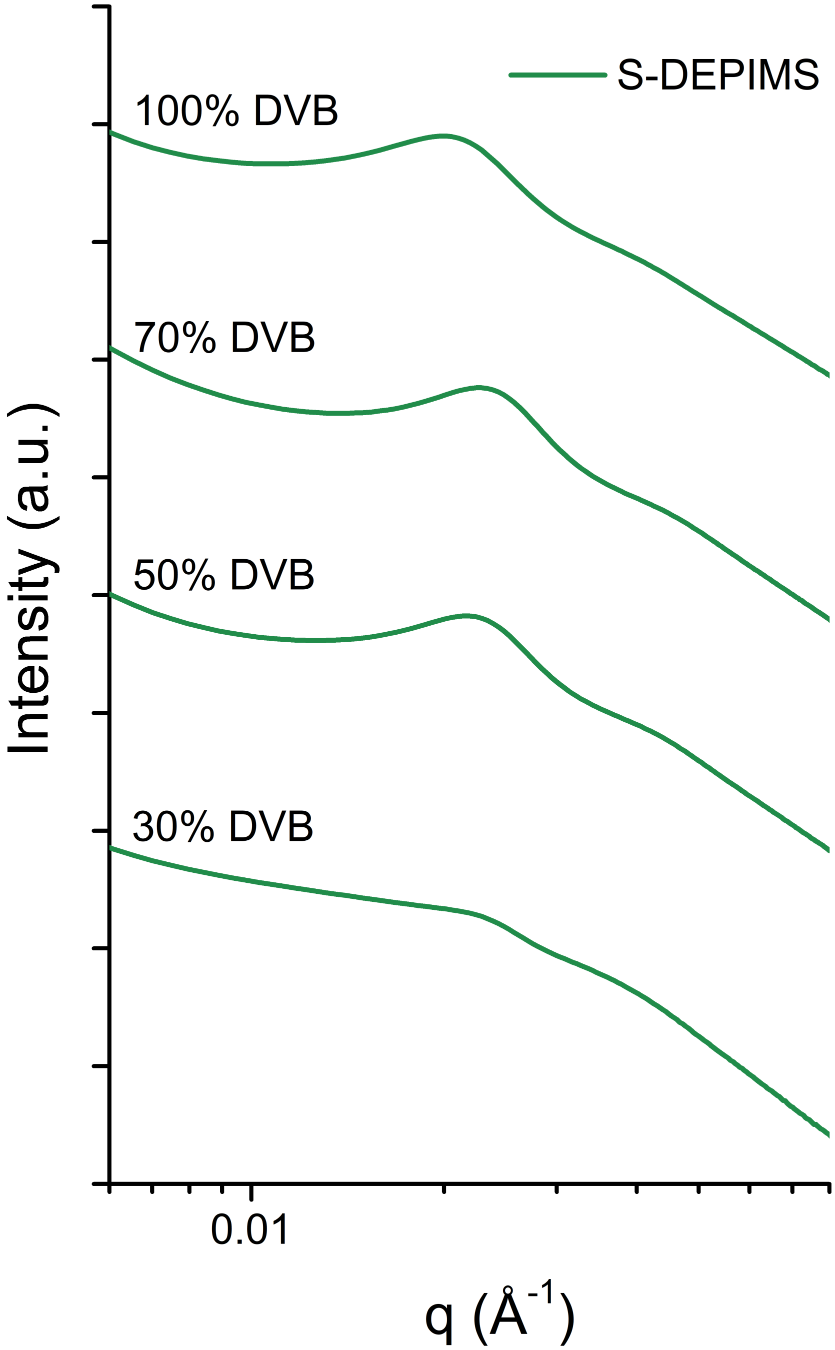

Figure S18. SAXS Scattering patterns for sulfonated DEPIMS (S-DEPIMS) at various crosslink densities demonstrating the retention of structure upon sulfonation. Scattering patterns are vertically shifted for clarity.

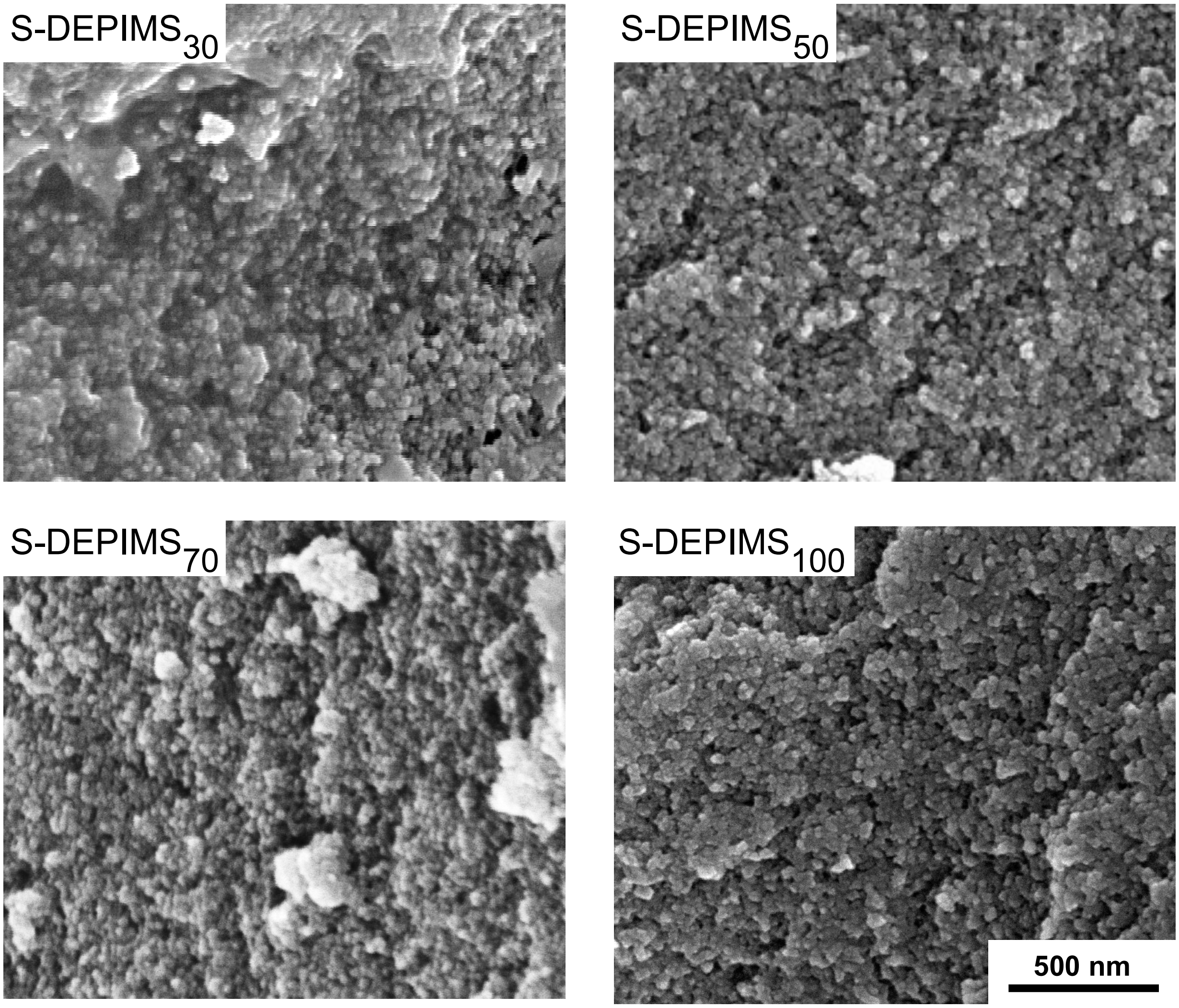

Figure S19. Scanning electron micrographs of S-DEPIMS materials demonstrating no significant morphology change upon sulfonation.

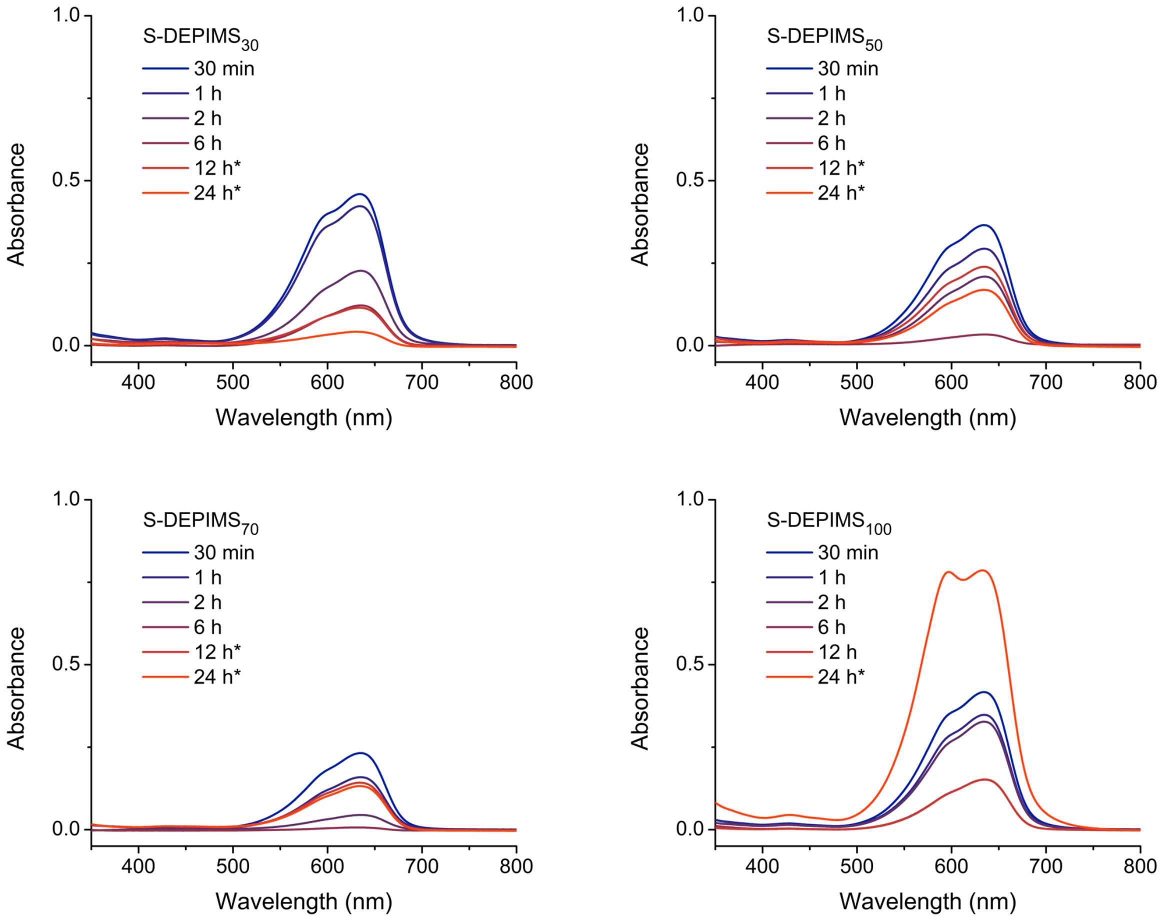

Figure S20. UV-Vis spectra of filtered Nile Blue solution after stirring with S-DEPIMS materials for the indicated amount of time shown in Figure 5E. All solutions were diluted by a factor of 10, except for timepoints labeled with *, which were undiluted.

Table S2. Absorption modeling parameters from Figure 5F and Figure S17. Q_{eq}, K_1, are equilibrium uptake and first order rate constants, respectively. The experimental dye uptake values were fit to the equation $Q = Q_{eq}(1 - e^{-K_1 t})$ where Q is uptake and t is time in hours via linear least-squares regression. Quality of fit is represented by R^2 values.

Sample	Q_{eq} (mg/g)	K_1 (h^{-1})	R^2
S-DEPIMS$_{30}$	298	0.280	0.97
S-DEPIMS$_{50}$	278	0.546	0.99
S-DEPIMS$_{70}$	293	1.40	0.97
S-DEPIMS$_{100}$	232	0.380	0.95

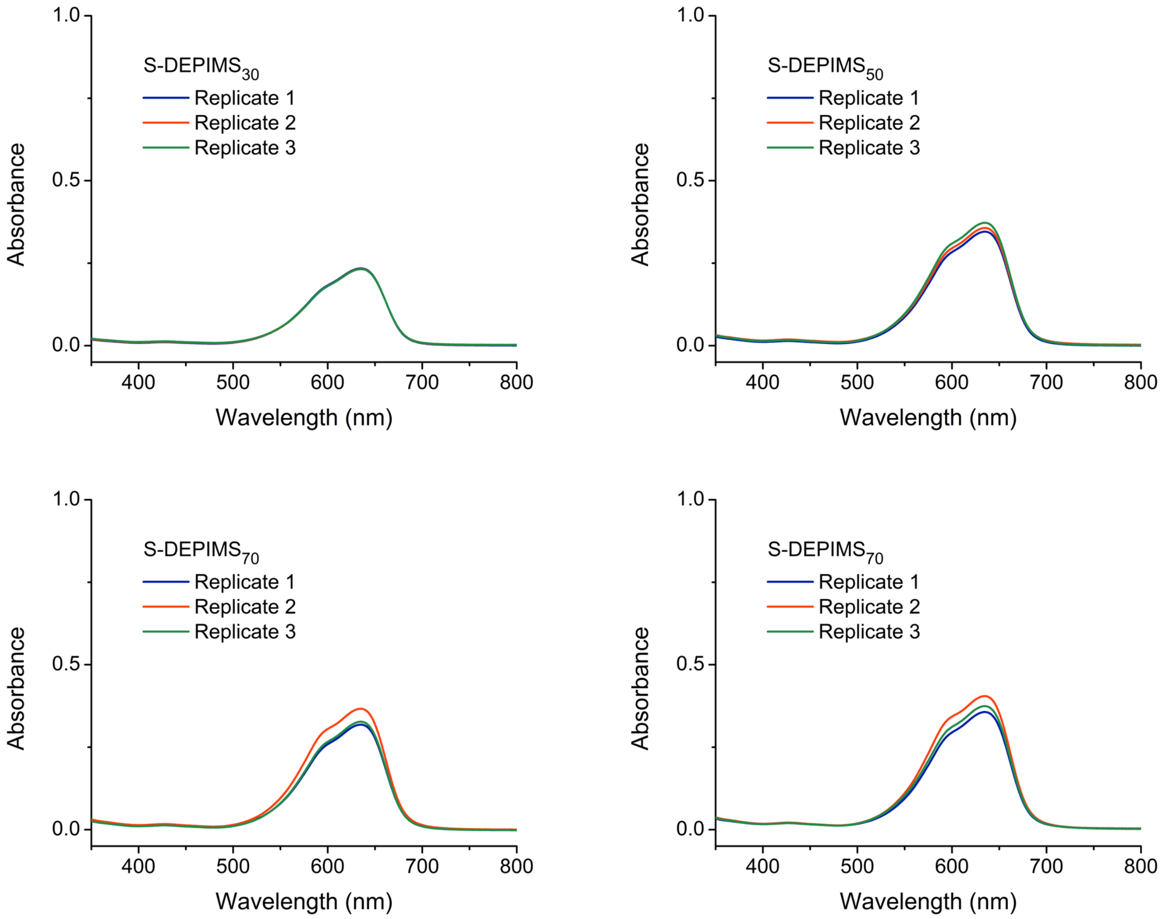

Figure S21. UV-Vis spectra of Nile Blue solutions demonstrating the uptake capacity of S-DEPIMS as shown in Figure 5F. All solutions are diluted by a factor of 10.

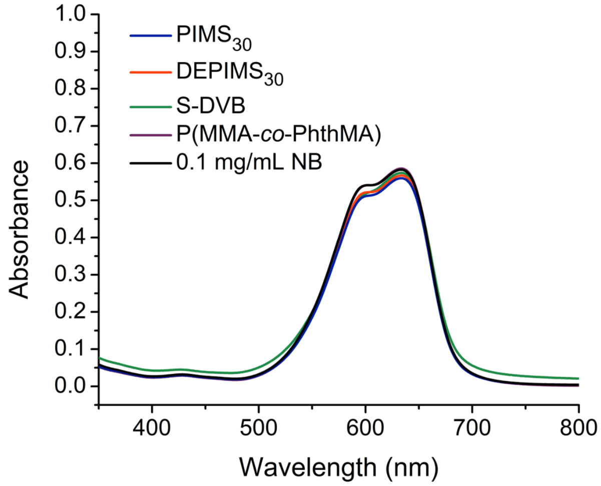

Figure S22. UV-Vis spectra of Nile Blue solutions demonstrating the negligible uptake capacity of a variety of controls related to S-DEPIMS. In particular, the low Nile Blue uptake by $DEPIMS_{30}$ and sulfonated crossliked divinylbenzene networks (S-DVB) indicates that the functionality and mesoporous architecture of S-DEPIMS is essential for their exceptional dye uptake behavior. All solutions are diluted by a factor of 10.

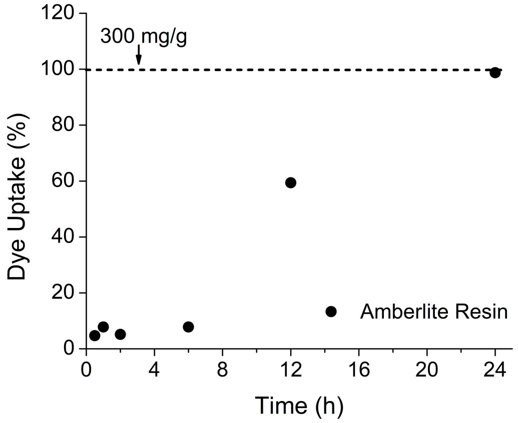

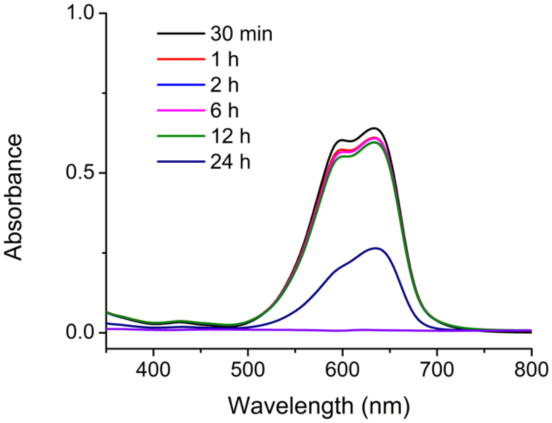

Figure S23. (Left) Kinetics of dye uptake upon stirring 15 mL of NB stock solution and 5 mg of commercial resin Amberlite IRC 120 for pre-determined times. (Right) UV-Vis spectra of filtered Nile Blue solution after stirring with S-DEPIMS materials for the indicated amount of time shown in Figure 5E. All solutions were diluted by a factor of 10.

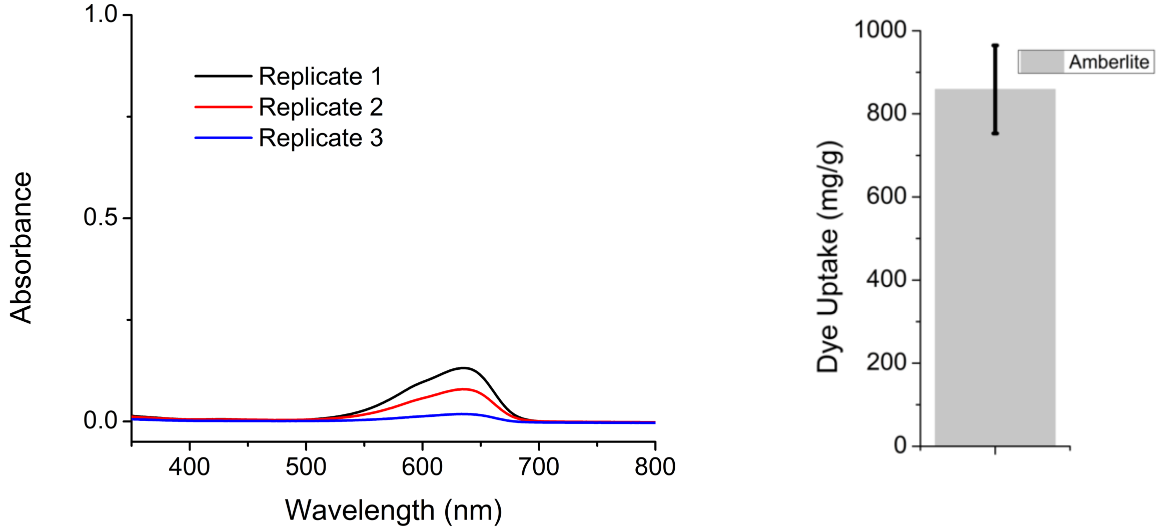

Figure S24. (Left) UV-Vis spectra of Nile Blue solutions demonstrating the uptake capacity of commercial resin Amberlite IRC 120. All solutions are diluted by a factor of 10. (Right) Uptake capacity of Amberlite IRC 120 in mg/g.

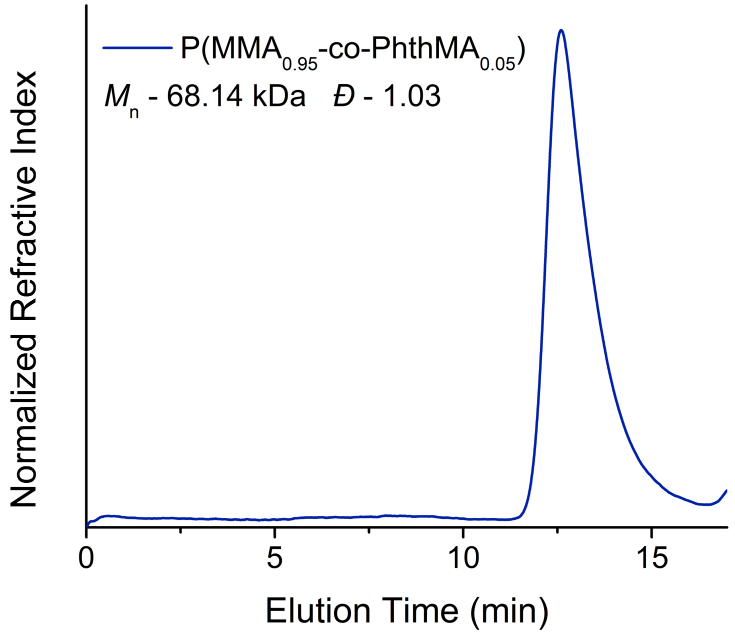

Figure S25. SEC-MALS of P($MMA_{0.95}$-*co*-$PhthMA_{0.05}$).

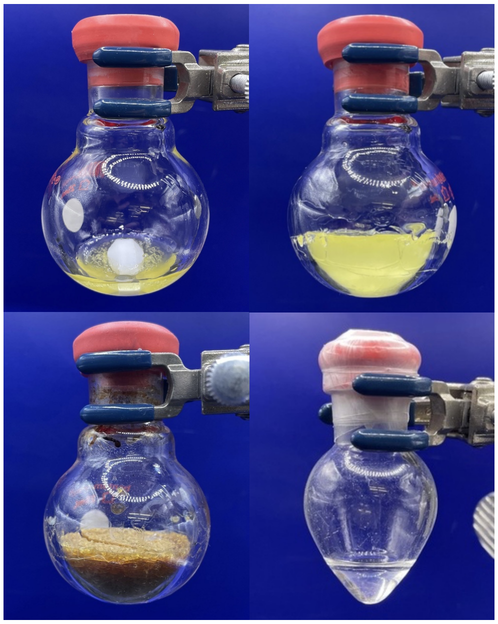

Figure S26. Photographs of (top left) macroCTA generated via bulk RAFT polymerization of MMA and PhthMA, (top right) PIMS generated by chain extension of the macroCTA with Sty and DVB, (bottom left) bulk DEPIMS materials ($DEPIMS_B$) generated via depolymerization in a distillation apparatus, and (bottom right) the distillate collected from the bulk $DEPIMS_B$ materials.

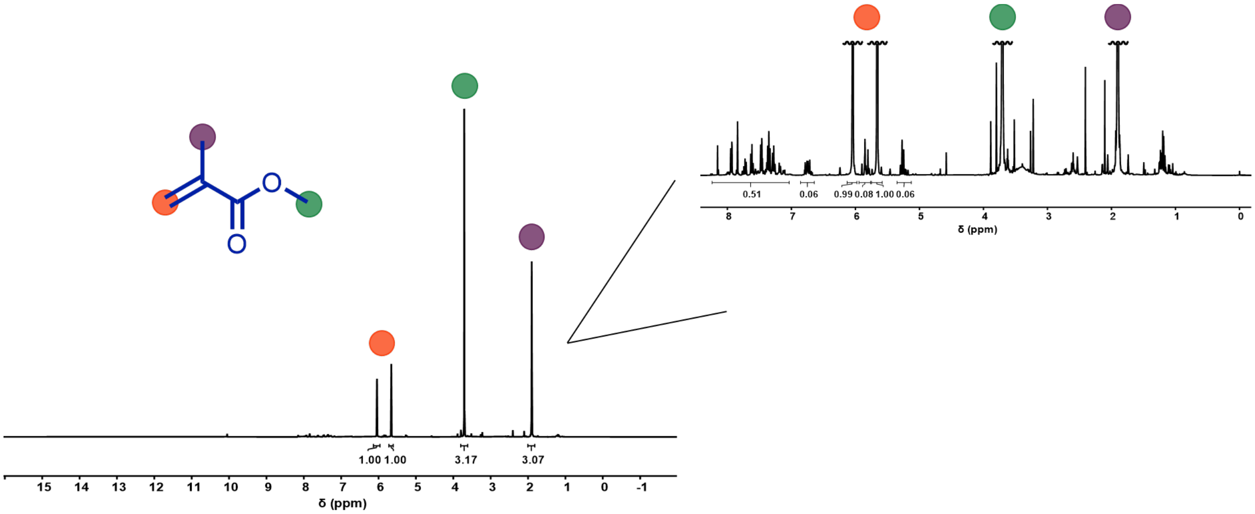

Figure S27. ^{1}H NMR spectrum of the distillate collected from the $DEPIMS_B$ materials. The distillate is primarily MMA, with a small percent of various aromatics we attribute to Sty and other monofunctional impurities present in technical grade DVB.

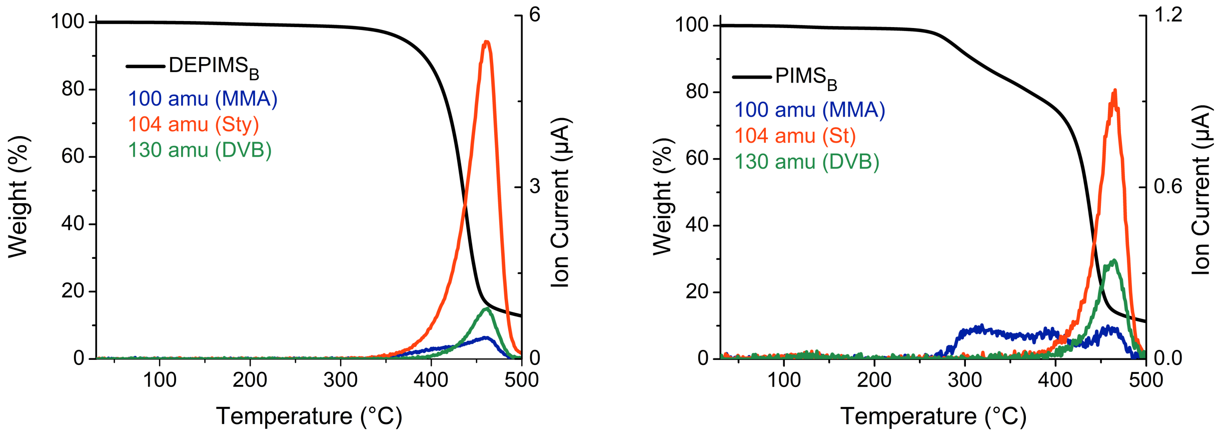

Figure S28. (Left) TGA-MS analysis of PIMS$_B$ materials ramped to 500 °C at 5 °C/min and (right) DEPIMS$_B$ materials ramped to 500 °C at 5 °C/min after being held at 290 °C for 15 min. MMA, Sty, and DVB are indicated by the blue, orange, and green lines, respectively. Compared to DEPIMS materials generated in a stepwise fashion, the bulk materials exhibit reduced efficiency of depolymerization, which we attribute to residual MMA creating indistinct phase boundaries during the PIMS process.

Table S3. BET surface areas estimated from nitrogen porosimetry at 77 K as shown in Figure 6E. Estimated from p/p_o of 0.05 to 0.035. Goodness of fit indicated by R^2.

Sample	BET Surface Area (m^2/g)	R^2
S-DEPIMS$_D$	141	0.9999

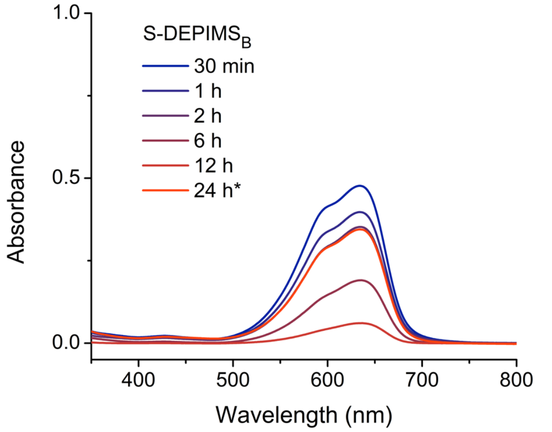

Figure S29. UV-Vis spectra of Nile Blue solutions demonstrating the uptake capacity of S-DEPIMS$_B$ as shown in Figure 6F. All solutions are diluted by a factor of 10, except those marked with *, which are undiluted.

Table S4. Absorption modeling parameters from Figure 6F and Figure S24. Q_{eq}, K_1, are equilibrium uptake and first order rate constants, respectively. The experimental dye uptake values were fit to the equation $Q = Q_{eq}(1 - e^{-K_1 t})$ where Q is uptake and t is time in hours via linear least-squares regression. Goodness of fit is represented by R^2 values.

Sample	Q_{eq} (mg/g)	K_1 (h^{-1})	R^2
S-DEPIMS$_B$	284.1	0.193	0.99

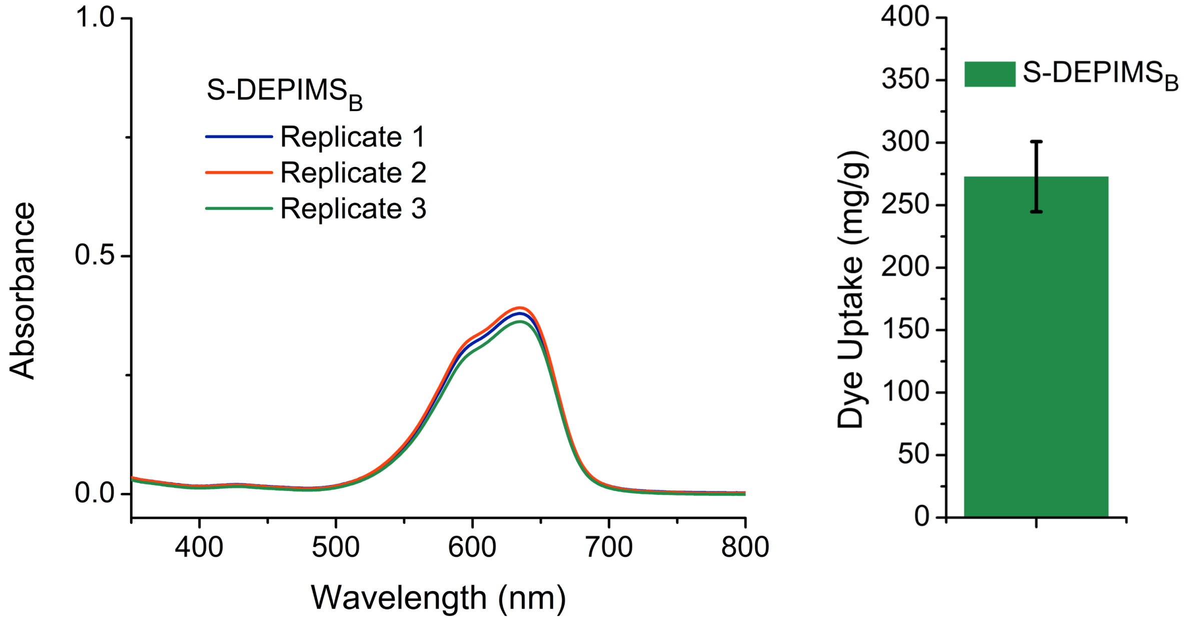

Figure S30. (Left) UV-Vis spectra of Nile Blue solutions demonstrating the uptake capacity of S-DEPIMS$_B$. All solutions are diluted by a factor of 10. (Right) Uptake capacity of S-DEPIMS$_B$ in mg/g.

S5. References

(1) Moad, G.; Chong, Y.; Postma, A.; Rizzardo, E.; Thang, S. H. Advances in RAFT polymerization: the synthesis of polymers with defined end-groups. *Polymer* **2005**, *46*, 8458–8468.

(2) Hughes, R. W.; Lott, M. E.; Zastrow, I. S.; Young, J. B.; Maity, T.; Sumerlin, B. S. Bulk depolymerization of methacrylate polymers via pendent group activation. *Journal of the American Chemical Society* **2024**, *146*, 6217–6224.

(3) Stevens, K. C.; Tirrell, M. V. Structure and properties of bottlebrush polyelectrolyte complexes. *Journal of Polymer Science* **2024**, *62*, 3808–3817.
